# MF-IEKF: A Multiplicative Federated Invariant Extended Kalman Filter for INS/GNSS

**DOI:** 10.3390/s26010127

**Published:** 2025-12-24

**Authors:** Lebin Zhao, Tao Chen, Peipei Yuan, Xiaoyang Li, Yang Luo

**Affiliations:** 1 School of Automobile, Chang’an University, Xi’an 710018, China; 2 Sichuan Chengneiyu Expressway Co., Ltd., Neijiang 641100, China

**Keywords:** multiplicative federated invariant EKF (MF-IEKF), inertial navigation system (INS), global navigation satellite system (GNSS), state estimation

## Abstract

**Highlights:**

**What are the main findings?**

- Based on the two left-invariant errors on the SE_2_(3) Lie group, a corrected error on the Lie algebra is proposed for the multiplicative corrected left-invariant extended Kalman filter (MCL-IEKF).
- Leveraging the decoupling of position and attitude in the right-invariant observation matrix of GNSS, a multiplicative federated IEKF (MF-IEKF) is developed by combining left- and right-invariant errors.

**What are the implications of the main findings?**

- MCL-IEKF improves state estimation accuracy compared to classical multiplicative left- (right-)IEKF.
- Under large misalignment angle conditions, MF-IEKF achieves faster convergence in position and velocity compared to the MCL-IEKF.

**Abstract:**

The integration of an inertial navigation system (INS) with the Global Navigation Satellite System (GNSS) is crucial for suppressing the error drift of the INS. However, traditional fusion methods based on the extended Kalman filter (EKF) suffer from geometric inconsistency, leading to biased estimates—a problem markedly exacerbated under large initial misalignment angles. The invariant extended Kalman filter (IEKF) embeds the state in the Lie group SE_2_(3) to establish a more consistent framework, yet two limitations remain. First, its standard update fails to synergize complementary error information within the left-invariant formulation, capping estimation accuracy. Second, velocity and position states converge slowly under extreme misalignment. To address these issues, a multiplicative federated IEKF (MF-IEKF) was proposed. A geometrically consistent state propagation model on SE_2_(3) is derived from multiplicative IMU pre-integration. Two parallel, mutually inverse left-invariant error sub-filters (ML_1_-IEKF and ML_2_-IEKF) cooperate to improve overall accuracy. For large-misalignment scenarios, a short-term multiplicative right-invariant sub-filter is introduced to suppress initial position and velocity errors. Extensive Monte Carlo simulations and KITTI dataset experiments show that MF-IEKF achieves higher navigation accuracy and robustness than ML_1_-IEKF.

## 1. Introduction

Inertial navigation systems (INSs) are indispensable in aerospace, autonomous driving, and robotics, owing to their full autonomy, high-rate output, and provision of complete motion information [1,2,3]. However, propagating attitude, velocity, and position through temporal integration of gyroscope and accelerometer data leads to the inherent accumulation and divergence of navigation errors, resulting in significant positional drift [4,5,6]. To suppress this drift and enhance accuracy, multi-sensor fusion, especially with the Global Navigation Satellite System (GNSS), has become indispensable.

Among existing fusion algorithms, frameworks based on the Extended Kalman Filter (EKF) [7,8] and its Error-State Kalman Filter (ESKF) variant [9,10] remain the most widely adopted. Nevertheless, these conventional methods suffer from fundamental limitations. Conventional methods model position and velocity in Euclidean space while representing attitude on the Lie group SO(3) or with quaternions. This mismatch in parameterization disrupts the intrinsic geometric structure. When state estimates deviate significantly, the local linearization employed by EKF/ESKF introduces substantial errors, jeopardizing filter convergence [11]. More critically, these methods exhibit geometric inconsistency [12]: the linearization process during measurement updates can induce spurious information gain in unobservable directions [13,14], thereby corrupting the state estimate. This phenomenon is well-established in Simultaneous Localization and Mapping (SLAM) [15,16,17] and visual-inertial odometry (VIO) systems [18,19].

To address this fundamental issue of geometric inconsistency, the invariant filtering framework, rooted in Riemannian manifold and Lie group theory, has emerged [20,21,22,23,24]. A key realization of this framework is the invariant extended Kalman filter (IEKF), which constructs a geometrically consistent filtering structure by embedding the entire system state into the matrix Lie group SE_2_(3). In this setting, the motion model satisfies the group-affine property [25], which results in error dynamics governed by a linear autonomous differential equation, independent of the true state trajectory [22]. This structural decoupling enables the IEKF to theoretically overcome the convergence pitfalls of traditional EKF methods that stem from linearization-point dependence. Consequently, the IEKF maintains superior estimation accuracy and long-term stability, even under large initial misalignment [26,27].

The IEKF can be categorized into the left-IEKF (L-IEKF) and the right-IEKF (R-IEKF) according to the error definition. The system matrix of L-IEKF is completely independent of the state trajectory, often leading to higher estimation accuracy, whereas that of R-IEKF excludes rapidly varying states, yielding superior temporal stability [20]. Consequently, the observation model must be aligned with either the left- or right-invariant form depending on its physical nature (global or local) [28]. This alignment is crucial for maintaining filter consistency throughout the process and for achieving optimal estimation [28,29,30,31]. Guided by this principle, IEKF has been successfully applied in globally referenced inertial-integrated navigation [30,32] and locally referenced VIO systems [21,33,34,35].

Recognizing the complementary advantages of left- and right-invariant models, researchers have begun to explore their joint utilization. For instance, Ref. [31] proposed a federated IEKF that processes left- and right-invariant observation models in parallel and dynamically selects the output according to the scenario, representing a preliminary exploration of multi-model collaboration. However, the intrinsic synergistic mechanisms between these error models were not thoroughly analyzed. In contrast, Ref. [28] established a connection between left- and right-invariant errors for attitude estimation using the adjoint matrix of the Lie group, improving accuracy. Yet, this collaborative framework was not extended to full state estimation encompassing velocity and position. These works indicate that exploring a deep fusion of left- and right-invariant models is a clear and valuable direction.

Despite initial attempts at fusion architectures, existing Lie group-based navigation methods still face two fundamental challenges in delivering high-precision, high-robustness performance. The first is the challenge of model fidelity and generalizability. Many studies rely on simplified models, such as assuming a flat Earth and neglecting Coriolis effects [36], which deviate from real-world navigation conditions. To address this, Ref. [36] proposed an equivariant filtering framework that accounts for Coriolis forces and introduced two new left- and right-invariant error models. Recent research strives to enhance geometric consistency under more realistic models, e.g., constructing precise kinematics and error models on the SE(3) group in the Earth-centered inertial (ECI) or Earth-centered, Earth-fixed (ECEF) reference frames [37,38]. These works deepen the theoretical foundation and improve attitude—particularly heading—estimation under large misalignment angles. However, their focus remains on refining the model within a single reference frame and on theoretical analysis. The second challenge is computational efficiency and structural consistency. Although many methods define states on Lie groups, their kinematic equations often adopt decoupled computational propagation [21,28,29,30,31,32,33,34,35,39], offering computational efficiency comparable to that of the traditional EKF without fully leveraging the structural advantages of Lie group methods. To address this, Ref. [40] introduced an auxiliary velocity and unified state propagation on the SE_2_(3) group, proposing an efficient multiplicative pre-integration theory that was later generalized into a unified mathematical framework by [41]. Ref. [42] applied this approach to INS/GNSS integration but did not effectively mitigate the dramatic growth of velocity and position errors caused by large misalignment angles during initial alignment. Recently, Ref. [43] proposed an Iterative Equivariant Filter (I-EqF) that enhances the robustness of tightly coupled SINS/GNSS navigation under nonlinearities and large misalignment through iterative updates and covariance resetting, representing progress in optimizing a single-filter algorithm.

Building on the aforementioned developments, current research has advanced invariant filtering technology along several fronts: model construction has become more rigorous [36,37,38]; numerical methods for single filters have been refined [43]; and valuable attempts have been made to fuse left- and right-invariant models [28,31]. Yet a unified framework that organically integrates two key considerations remains elusive: at the architectural level, how to transcend simple parallel or switching mechanisms to achieve deep synergy between left- and right-invariant error estimations;, in the presence of large initial misalignment, how to exploit the right-invariant model’s insensitivity to attitude error to accelerate the convergence of velocity and position states. Addressing these challenges motivates the present work.

**Figure 1:**
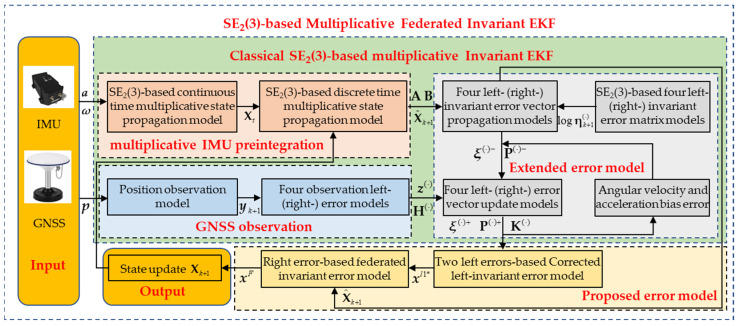
The proposed multiplicative federated invariant EKF framework. ^(⋅)^ represents the different error models.

To synergistically leverage the complementary strengths of different invariant error models and effectively handle large initial misalignment, we propose a novel multiplicative federated IEKF (MF-IEKF) framework as shown in Figure 1. The core contribution is an architectural innovation that fuses the advantages of multiple invariant models through a deep synergistic mechanism. Specifically:

1. Leveraging multiplicative pre-integration theory [40,41], we derive a state propagation equation that evolves on the SE_2_(3) Lie group, ensuring geometric consistency and computational efficiency.
2. Adopting the four invariant error models proposed in [36], we construct two parallel left-invariant error sub-filters using GNSS observations. By exploiting the inverse relationship between their error estimates, we perform collaborative correction that significantly improves steady-state accuracy.
3. During the initial phase, we introduce a multiplicative right-invariant sub-filter that exploits the attitude-error decoupling property of right-invariant observation models to accelerate velocity and position convergence, thereby effectively mitigating the slow initial convergence exhibited by left-invariant models under large misalignment.
4. Rigorous validation via extensive Monte Carlo simulations and experiments on public datasets demonstrates the efficacy of the proposed MF-IEKF. Compared with the multiplicative left-IEKF (ML_1_-IEKF) baseline, our method achieves consistent improvements of at least 5.26% in attitude, 8.72% in velocity, and 15.28% in position estimation.

## 2. IMU Model on Lie Group

This section develops a linearized inertial measurement unit (IMU) kinematic model within the Earth-Centered Earth-Fixed (ECEF) reference frame. The following coordinate systems are formally defined: e-frame (ECEF), b-frame (body-fixed coordinate system), i-frame (inertial reference frame), and n-frame (local navigation frame).

### 2.1. IMU-Based Kinematic Equations

The kinematic difference equations for the attitude, velocity, and position in the e-frame are given by

(1) R˙be=Rbeωibb−∧ωiee∧Rbe

(2) v˙ebe=Rbeaibb−2ωieevebe∧+gibe

(3) $${\dot{p}}_{eb}^{e}=v_{eb}^{e}$$

where $R_{b}^{e}\in {\mathbb{R}}^{3\times 3}$ is the attitude matrix from b-frame to e-frame; the symbol ${\left(\cdot \right)}_{eb}^{e}$ in the velocity $v_{eb}^{e}$ and position $p_{eb}^{e}$ denotes the b-frame with respect to the e-frame in the e-frame; $a_{ib}^{b}$ and $\omega_{ib}^{b}\in {\mathbb{R}}^{3\times 1}$ represent the acceleration and angular rate relative to the i-frame expressed in the b-frame; $\omega_{ie}^{e}\in {\mathbb{R}}^{3\times 1}$ is the angular rate of the Earth rotation in the e-frame; $g_{ib}^{e}$ is the gravity vector. The term −2ωieevebe∧ are so called Coriolis acceleration, where the symbol ${\left(\cdot \right)}^{\wedge }\in {\mathbb{R}}^{3\times 3}$ is the skew-symmetric matrix operator. For a vector $\omega ={\left[\omega_{x},\omega_{y},\omega_{z}\right]}^{T}$, its skew-symmetric matrix is defined as:

(4) $$\omega^{\wedge }=\left[\begin{array}{ccc} 0 & -\omega_{z} & \omega_{y}\\ \omega_{z} & 0 & -\omega_{x}\\ -\omega_{y} & \omega_{x} & 0 \end{array}\right].$$

To establish a unified framework on the manifold, the paper employs an auxiliary velocity [40].

(5) vibe=vebe+ωieepibe∧

where $p_{ib}^{e}$ is the b-frame with respect to the i-frame in the e-frame. Given the colocation of origins between the e-frame and i-frame, there exists the equivalence relation $p_{ib}^{e}=p_{eb}^{e}$. Based on (3) and (5), the differential equation of $p_{ib}^{e}$ can be written as

(6) p˙ibe=p˙ebe=vebe=vibe−ωieepibe∧

$R_{b}^{e}$, $v_{ib}^{e}$ and $p_{ib}^{e}$ are embedded into the matrix Lie group ${\mathrm{SE}}_{2}(3)$ [22], the state **X** can be represented as

(7) $$X=\left[\begin{array}{ccc} R_{b}^{e} & v_{ib}^{e} & p_{ib}^{e}\\ 0_{1\times 3} & 1 & 0\\ 0_{1\times 3} & 0 & 1 \end{array}\right]\in {\mathbb{R}}^{5\times 5}$$

Based on (1)–(6), the kinematic difference equation of state matrix **X** can be rewritten as

(8) X˙=g(X)=R˙bev˙ibep˙ibe02×302×2=Rbeωibb−∧ωiee∧RbeRbeaibb−ωieevibe∧+Gibevibe−ωieepibe∧02×302×2

where Gibe=(ωiee)∧2pibe+gibe is the gravitational vector, it can be derived through vector superposition of centrifugal acceleration with the gravity vector [36].

It is easily verified that (8) can be decomposed to [25]

(9) g(X)=XU+f(X)+WX

where the matrix **U** is related to the input, and the form is

(10) $$U=\left[\begin{array}{cc} {\omega_{ib}^{b}}^{\wedge } & \begin{array}{cc} a_{ib}^{b} & 0_{3\times 1} \end{array}\\ 0_{2\times 3} & 0_{2\times 2} \end{array}\right]$$

The matrix **W** is with respect to the Earth rotation and gravity vector in the e-frame, and it has

(11) $$W=\left[\begin{array}{cc} -{\omega_{ie}^{e}}^{\wedge } & \begin{array}{cc} G_{ib}^{e} & 0_{3\times 1} \end{array}\\ 0_{2\times 3} & 0_{2\times 2} \end{array}\right]$$

f(⋅) is a drift field related to velocity, as defined by

(12) $$f:X=\left[\begin{array}{ccc} R_{b}^{e} & v_{ib}^{e} & p_{ib}^{e}\\ 0_{1\times 3} & 1 & 0\\ 0_{1\times 3} & 0 & 1 \end{array}\right]\mapsto f(X)=\left[\begin{array}{cc} 0_{3\times 3} & \begin{array}{cc} 0_{3\times 1} & v_{ib}^{e} \end{array}\\ 0_{2\times 3} & 0_{2\times 2} \end{array}\right]$$

It can be easily checked that g(X) has group-affine property, and satisfies the following equation

(13) $$g(X_{1}X_{2})=g(X_{1})X_{2}+X_{1}g(X_{2})-X_{1}g(I_{5\times 5})X_{2}$$

where $X_{1},X_{2}\in {\mathrm{SE}}_{2}(3)$ are the implementation of (6), and $I_{5\times 5}$ is the group identity element in the ${\mathrm{SE}}_{2}(3)$. Obviously, f(⋅) also has the group-affine property

(14) $$f(X_{1}X_{2})=f(X_{1})X_{2}+X_{1}f(X_{2})-X_{1}f(I_{5\times 5})X_{2}$$

since $f\left(I_{5\times 5}\right)=0_{5\times 5}$, (23) can be updated as

(15) $$f(X_{1}X_{2})=f(X_{1})X_{2}+X_{1}f(X_{2})$$

### 2.2. Decomposition of IMU State Matrix on the Li Group in Continuous Time

As the group affine property of the f(X), the state matrix **X** at arbitrary time *t* in the e-frame can be decomposed into a product function of the initial state $X_{0}$ as [40]

(16) $$X_{t}=L_{t}\Phi_{t}(X_{0})\Upsilon_{t}$$

where the concrete definition of $\Phi_{t}(\cdot )$ can be given as [40]

(17) $$\Phi_{t}:X=\left[\begin{array}{ccc} R_{b}^{e} & v_{ib}^{e} & p_{ib}^{e}\\ 0_{1\times 3} & 1 & 0\\ 0_{1\times 3} & 0 & 1 \end{array}\right]\mapsto \left[\begin{array}{ccc} R_{b}^{e} & v_{ib}^{e} & p_{ib}^{e}+tv_{ib}^{e}\\ 0_{1\times 3} & 1 & 0\\ 0_{1\times 3} & 0 & 1 \end{array}\right]=X+t\cdot f(X)$$

Obviously, $\Phi_{t}(\cdot )$ only depends on the time *t*. It is easy to verify the group automorphism property

(18) $$\Phi_{t}(X_{1}X_{2})=\Phi_{t}(X_{1})\Phi_{t}(I_{5\times 5})\Phi_{t}(X_{2})=\Phi_{t}(X_{1})\Phi_{t}(X_{2})$$

According to the definition of -, it can be concluded that $\Phi_{t}(I_{5\times 5})=I_{5\times 5}$.

The concrete form of $L_{t}$ in (16) can be given as

(19) $$L_{t}=\left[\begin{array}{ccc} L_{t}^{R} & L_{t}^{v} & L_{t}^{p}\\ 0_{1\times 3} & 1 & 0\\ 0_{1\times 3} & 0 & 1 \end{array}\right]$$

Simultaneously, $L_{t}$ is the solution of differential Equation (20)

(20) $${\dot{L}}_{t}=\left[\begin{array}{ccc} -{\omega_{ie}^{e}}^{\wedge }L_{t}^{R} & G_{ib}^{e}-{\omega_{ie}^{e}}^{\wedge }L_{t}^{v} & L_{t}^{v}-{\omega_{ie}^{e}}^{\wedge }L_{t}^{p}\\ 0_{1\times 3} & 1 & 0\\ 0_{1\times 3} & 0 & 1 \end{array}\right]=WL_{t}+f(L_{t})$$

where **W** is defined in (11). Based on the differential equation in (20), $L_{t}$ represents the global increment in the e-frame that undergoes slow variation due to the action of the Earth rotation. In addition, the initial value is $L_{0}=I_{5\times 5}$.

Meanwhile, the concrete form of $\Upsilon_{t}$ in (16) can be written as

(21) $$\Upsilon_{t}=\left[\begin{array}{ccc} \Upsilon_{t}^{R} & \Upsilon_{t}^{v} & \Upsilon_{t}^{p}\\ 0_{1\times 3} & 1 & 0\\ 0_{1\times 3} & 0 & 1 \end{array}\right]$$

and $\Upsilon_{t}$ is the solution of the differential Equation (22)

(22) $${\dot{\Upsilon }}_{t}=\left[\begin{array}{ccc} \Upsilon_{t}^{R}{\omega_{ib}^{b}}^{\wedge } & \Upsilon_{t}^{R}a_{ib}^{b} & \Upsilon_{t}^{v}\\ 0_{1\times 3} & 1 & 0\\ 0_{1\times 3} & 0 & 1 \end{array}\right]=U\Upsilon_{t}+f(\Upsilon_{t})$$

where **U** is defined in (10). Based on (22), $\Upsilon_{t}$ is associated with the high-frequency input from the IMU, namely the acceleration $a_{ib}^{b}$ and angular velocity $\omega_{ib}^{b}$. It reflects the local increment during the motion process in the b-frame, and it can be used to pre-integrate. Similarly to $L_{0}$, the initial value is also $\Upsilon_{0}=I_{5\times 5}$. It should be noted that both $L_{t}$ and $\Upsilon_{t}$ depend on the initial state $X_{0}$.

The state matrix $X_{t}$ in (16) can be expanded as

(23) $$X_{t}=\left[\begin{array}{ccc} L_{t}^{R}R_{0}\Upsilon_{t}^{R} & L_{t}^{R}(R_{0}\Upsilon_{t}^{v}+v_{0})+L_{t}^{v} & L_{t}^{R}(R_{0}\Upsilon_{t}^{p}+p_{0}+tv_{0})+L_{t}^{p}\\ 0_{1\times 3} & 1 & 0\\ 0_{1\times 3} & 0 & 1 \end{array}\right]$$

where $R_{0}$, $v_{0}$ and $p_{0}$ with their frame notations omitted here due to space constraints, correspond to $R_{b,0}^{e}$, $v_{eb,0}^{e}$ and $p_{eb,0}^{e}$, separately.

Hereby, the paper establishes the equations for the continuous-time state matrix $X_{t}$ in the e-frame.

### 2.3. The State Matrix on Li Group in Discrete Time

In practical implementations, state estimation systems inherently process discretized sensor inputs, such as high-frequency acceleration and angular velocity measurements sampled at discrete intervals. This operational constraint necessitates the discretization of the continuous formulation presented in (16).

The state $X_{t}$ at the time *k* + 1 can be given as

(24) $$X_{k+1}=L_{k+1}\Phi_{k+1}(X_{0})\Upsilon_{k+1}$$

where $L_{k+1}$ and $\Upsilon_{k+1}$ were defined in (19) and (21). According to (17), $\Phi_{k+1}(X_{0})$ can be written as

(25) $$\Phi_{k+1}(X_{0})=\left[\begin{array}{ccc} R_{0} & v_{0} & p_{0}+t_{k}v_{ib}^{e}\\ 0_{1\times 3} & 1 & 0\\ 0_{1\times 3} & 0 & 1 \end{array}\right]$$

The initial objective focuses on deriving the temporal evolution of the global increment $L_{k}$ across consecutive time steps. Through discretization of the continuous formulation in (20), the discrete-time representations of global increments $L_{k+1}^{R}$, $L_{k+1}^{v}$, and $L_{k+1}^{p}$ at discrete time $t_{k+1}$ are expressed through the following relationships

(26) $$L_{k+1}=L_{\Delta t}\Phi_{\Delta t}(L_{k})$$

where

(27) $$L_{\Delta t}=\left[\begin{array}{ccc} L_{\Delta t}^{R} & L_{\Delta t}^{v} & L_{\Delta t}^{p}\\ 0_{1\times 3} & 1 & 0\\ 0_{1\times 3} & 0 & 1 \end{array}\right]$$

(28) $$\Phi_{\Delta t}(L_{k})=\left[\begin{array}{ccc} L_{k}^{R} & L_{k}^{v} & \Delta tL_{k}^{v}+L_{k}^{p}\\ 0_{1\times 3} & 1 & 0\\ 0_{1\times 3} & 0 & 1 \end{array}\right]$$

(29) $$\begin{array}{c} L_{\Delta t}^{R}=\Gamma_{0}\left(-\omega_{ie}^{e}\Delta t\right)\\ L_{\Delta t}^{v}=\Gamma_{1}\left(-\omega_{ie}^{e}\Delta t\right)\Delta tG_{ib}^{e}\\ L_{\Delta t}^{p}=\Gamma_{2}\left(-\omega_{ie}^{e}\Delta t\right)\Delta t^{2}G_{ib}^{e} \end{array}$$

The detailed derivation of $L_{\Delta t}$ can be found in Appendix A. The definition of function $\Gamma_{m}\left(\varphi \right)$ is [29]

(30) $$\Gamma_{m}\left(\varphi \right)≜\sum_{n=0}^{\infty }\frac{1}{\left(n+m\right)!}{\left(\varphi^{\wedge }\right)}^{n},\varphi \in {\mathbb{R}}^{3\times 1},m=0,1,2\ldots$$

The above derivation quantifies the variation in the global increment across consecutive time intervals. Next, we now turn to the evolution of the local increment $\Upsilon_{t}$ over consecutive time intervals. Based on (22), $\Upsilon_{t}$ at time $t_{k+1}$ can be expressed as

(31) $$\Upsilon_{k+1}=\Phi_{\Delta t}(\Upsilon_{k})\Upsilon_{\Delta t}$$

where

(32) $$\Phi_{\Delta t}(\Upsilon_{k})=\left[\begin{array}{ccc} \Upsilon_{k}^{R} & \Upsilon_{k}^{v} & \Delta t\Upsilon_{k}^{v}+\Upsilon_{k}^{p}\\ 0_{1\times 3} & 1 & 0\\ 0_{1\times 3} & 0 & 1 \end{array}\right]$$

(33) $$\Upsilon_{\Delta t}=\left[\begin{array}{ccc} \Upsilon_{\Delta t}^{R} & \Upsilon_{\Delta t}^{v} & \Upsilon_{\Delta t}^{p}\\ 0_{1\times 3} & 1 & 0\\ 0_{1\times 3} & 0 & 1 \end{array}\right]$$

where $\Upsilon_{\Delta t}^{R}$, $\Upsilon_{\Delta t}^{v}$ and $\Upsilon_{\Delta t}^{p}$ are the local increments between two consecutive discrete-time intervals Δt, and based on (22), $\Upsilon_{\Delta t}$ can be obtained as

(34) $$\begin{array}{c} \Upsilon_{\Delta t}^{R}=\Gamma_{0}\left(\omega_{ib}^{b}\Delta t\right)\\ \Upsilon_{\Delta t}^{v}=\Gamma_{1}(\omega_{ib}^{b}\Delta t)\Delta ta_{ib}^{b}\\ \Upsilon_{\Delta t}^{p}=\Gamma_{2}(\omega_{ib}^{b}\Delta t)\Delta t^{2}a_{ib}^{b} \end{array}$$

The detailed derivation of $\Upsilon_{\Delta t}$ can be found in Appendix A, where $\Gamma_{m}\left(\varphi \right)$ was defined in (30); the inputs $a_{ib}^{b}$ and $\omega_{ib}^{b}$ are assumed to be constant during each discrete-time interval Δt and take the initial values at $t_{k}$. This assumption is reasonable and common when processing the IMU data.

By substituting (26) and (31) into (24), the discrete-time state equation can be derived as follows

(35) $$X_{k+1}=L_{\Delta t}\Phi_{\Delta t}\left(X_{k}\right)\Upsilon_{\Delta t}$$

Proof:

(36) $$\begin{array}{cc} X_{k+1} & =L_{k+1}\Phi_{k+1}(X_{0})\Upsilon_{k+1}\\ & =L_{\Delta t}\Phi_{\Delta t}(L_{k})\Phi_{k+\Delta t}(X_{0})\Phi_{\Delta t}(\Upsilon_{k})\Upsilon_{\Delta t}\\ & =L_{\Delta t}\Phi_{\Delta t}(L_{k})\Phi_{\Delta t}(\Phi_{k}(X_{0}))\Phi_{\Delta t}(\Upsilon_{k})\Upsilon_{\Delta t}\\ & =L_{\Delta t}\Phi_{\Delta t}(L_{k}\Phi_{k}(X_{0})\Upsilon_{k})\Upsilon_{\Delta t}\\ & =L_{\Delta t}\Phi_{\Delta t}(X_{k})\Upsilon_{\Delta t} \end{array}$$

where $\Phi_{t}\left(X\right)$ possesses another cumulative property with respect to time and the proof is presented as following

(37) $$\begin{array}{cc} \Phi_{t_{i}}(\Phi_{t_{j}}(X)) & =\Phi_{t_{i}}(X+t_{j}f(X))\\ & =X+t_{j}f(X)+t_{i}f(X+f(X))\\ & =X+(t_{i}+t_{j})f(X)=\Phi_{t_{i}+t_{j}}(X) \end{array}$$

As demonstrated in (35), we have established the discrete time propagation equation for the state matrix X. This formulation exhibits intrinsic multiplicative properties that enable unified computation within the Lie group framework, in contrast to traditional EKFs that rely on additive error formulations. The updated state $X_{k+1}$ comprises three geometrically interpretable components:

1. Global increment $L_{\Delta t}$ induced by Earth’s rotation.
2. Prior state $\Phi_{\Delta t}(X_{k})$.
3. Local increment $\Upsilon_{\Delta t}$ generated by IMU high-frequency inputs.

## 3. Invariant Error State in Earth Frame

### 3.1. Four Invariant Errors in E-Frame

Similarly to the state matrix **X**, the error state is also defined on the Lie group ${\mathrm{SE}}_{2}(3)$.

**Definition** **1** (invariant error [22])**.** *The left- and right-invariant error matrices between the true state* $X\in {\mathrm{SE}}_{2}\left(3\right)$ *and estimation* $\hat{X}\in {\mathrm{SE}}_{2}\left(3\right)$ *are defined as*

*Right-invariant error matrix:*

(38) $$\eta^{r1}=\hat{X}X^{-1}$$

*Left-invariant error matrix:*

(39) $$\eta^{l1}=X^{-1}\hat{X}$$

Invariance Property: The term “invariant” signifies a key geometric property of these definitions. Consider an arbitrary fixed transformation $C\in {\mathrm{SE}}_{2}\left(3\right)$. If both states are transformed by the same left-multiplication (i.e., X→CX and $\hat{X}\to C\hat{X}$), the computed error remains unchanged.

**X** and the estimate $\hat{X}$ by the same transformation **C** does not alter the error. Alternative definitions:

Right-invariant error matrix:

(40) $$\eta^{r1}=\hat{X}X^{-1}=(C\hat{X}){\left(CX\right)}^{-1}$$

Left-invariant error matrix:

(41) $$\eta^{l1}=X^{-1}\hat{X}={\left(CX\right)}^{-1}(C\hat{X})$$

The introduction of the arbitrary matrix **C** in the above equalities is solely to demonstrate this invariance. It proves that the errors $\eta^{r1}$ and $\eta^{l1}$ depend only on the relative difference between $\hat{X}$ and **X**, and not on their absolute coordinates in the state space. This property is fundamental for the development of invariant filters, such as the MF-IEKF proposed in this work, which are known to exhibit improved convergence characteristics, especially under large misalignment conditions.

Exploiting this frame-invariance, one can equivalently define the errors inversely [30], leading to an alternative set of invariant errors:

Alternative right-invariant error matrix:

(42) $$\eta^{r2}=X{\hat{X}}^{-1}=(CX){\left(C\hat{X}\right)}^{-1}$$

Alternative left-invariant error matrix:

(43) $$\eta^{l2}={\hat{X}}^{-1}X={\left(C\hat{X}\right)}^{-1}(CX).$$

It is evident that error states $\eta^{r1}={\left(\eta^{r2}\right)}^{-1}$ and $\eta^{l1}={\left(\eta^{l2}\right)}^{-1}$ represent two opposing characterizations on the manifold.

### 3.2. Noise Model and Approximation

IMU measurements comprise three principal components: true kinematic values, sensor biases, and stochastic noise. These components can be mathematically modeled through the following

(44) $${\tilde{a}}_{ib}^{b}=a_{ib}^{b}+b_{a}+n_{a}$$

(45) $${\tilde{\omega }}_{ib}^{b}=\omega_{ib}^{b}+b_{\omega }+n_{\omega }$$

where $\tilde{a}$ and $\tilde{\omega }$ are the acceleration and gyroscope measurements expressed in the b-frame; $n_{a}$ and $n_{\omega }$ modeled as white Gaussian noise processes, satisfy $n_{a}∼N(0,\sigma_{a}^{2})$ and $n_{\omega }∼N(0,\sigma_{\omega }^{2})$, respectively; $b_{a}$ and $b_{\omega }$ denote the acceleration and gyroscope biases, whose temporal evolution follows Gaussian Markov processes.

(46) $$\dot{b}=\left[\begin{array}{c} {\dot{b}}_{a}\\ {\dot{b}}_{\omega } \end{array}\right]=\left[\begin{array}{cc} -\mathrm{diag}(\frac{1}{\tau_{a}}) & 0_{3\times 3}\\ 0_{3\times 3} & -\mathrm{diag}(\frac{1}{\tau_{\omega }}) \end{array}\right]\left[\begin{array}{c} b_{a}\\ b_{\omega } \end{array}\right]+\left[\begin{array}{c} n_{b_{a}}\\ n_{b_{\omega }} \end{array}\right]=F_{b}b+n_{b}$$

The noise in the IMU is not negligible, and the effect of such noise can be characterized as a right perturbation on the local increment

(47) $$\Upsilon_{\Delta t}={\hat{\Upsilon }}_{\Delta t}\exp(-N_{\Delta t})={\hat{\Upsilon }}_{\Delta t}\exp(-G_{k}n_{k})$$

where $N_{\Delta t}$ is the perturbation and satisfies $N_{\Delta t}∼N(0_{9\times 1},G_{k}\mathrm{cov}(n_{k})G_{k}^{T})$; $n_{k}={\left[\begin{array}{cc} n_{\omega }^{T} & n_{a}^{T} \end{array}\right]}^{T}$ defined in (44) and (45); and $G_{k}\in {\mathbb{R}}^{9\times 6}$ is the Jacobi matrix with respect to the $n_{k}$, and can be written as

(48) Gk=Γ0−1Γ1Δt03×3−Γ0−1Γ1a^ibbΓ2T∧Δt2Γ0−1Γ1Δt−Γ0−1Γ2a^ibbΓ3T∧Δt3Γ0−1Γ2Δt2

where the detailed derivation of $G_{k}$ can be found in Appendix B.

${\hat{\Upsilon }}_{\Delta t}$ corresponds to the local increment that encompasses uncertainty. Based on (34), ${\hat{\Upsilon }}_{\Delta t}$ can be expressed as

(49) $${\hat{\Upsilon }}_{\Delta t}=\left[\begin{array}{ccc} {\hat{\Upsilon }}_{\Delta t}^{R} & {\hat{\Upsilon }}_{\Delta t}^{v} & {\hat{\Upsilon }}_{\Delta t}^{p}\\ 0_{1\times 3} & 1 & 0\\ 0_{1\times 3} & 0 & 1 \end{array}\right]$$

where

(50) $$\begin{array}{c} {\hat{\Upsilon }}_{\Delta t}^{R}=\Gamma_{0}({\hat{\omega }}_{ib}^{b}\Delta t)\\ {\hat{\Upsilon }}_{\Delta t}^{v}=\Gamma_{1}({\hat{\omega }}_{ib}^{b}\Delta t){\hat{a}}_{ib}^{b}\Delta t\\ {\hat{\Upsilon }}_{\Delta t}^{p}=\Gamma_{2}({\hat{\omega }}_{ib}^{b}\Delta t){\hat{a}}_{ib}^{b}\Delta t^{2} \end{array}$$

where ${\hat{\omega }}_{ib}^{b}={\tilde{\omega }}_{ib}^{b}-b_{\omega }$, ${\hat{a}}_{ib}^{b}={\tilde{a}}_{ib}^{b}-b_{a}$.

### 3.3. Right- and Left-Invariant Error Kinematic Equations Based the Lie Algebra in Discrete Time

This section maps the error state matrices η in the Lie group ((40)–(43)) to Lie algebras $x={\left[\begin{array}{ccc} x_{\varphi }^{T} & x_{v}^{T} & x_{p}^{T} \end{array}\right]}^{T}\in {\mathbb{R}}^{9}$, where $x_{\varphi }$, $x_{v}$, $x_{p}$ correspond to the rotation, velocity, and position errors, respectively. These mappings exhibit the following relationship

(51) $$\eta =\exp(x)=\mathrm{Exp}(x^{\wedge })$$

Moreover, based on the definition of $\Phi_{t}(\cdot )$ in (26), the log-linearity property can be straightforwardly verified as

(52) $${\left(\Phi_{t}(X)\right)}^{-1}=\Phi_{t}(X^{-1})$$

and

(53) $$\Phi_{t}(\exp(x))=\Phi_{t}(\mathrm{Exp}(x^{\wedge }))=\mathrm{Exp}(F_{t}x)$$

where $F_{t}$ can be written as

(54) $$F_{t}=\left[\begin{array}{ccc} I_{3\times 3} & 0_{3\times 3} & 0_{3\times 3}\\ 0_{3\times 3} & I_{3\times 3} & 0_{3\times 3}\\ 0_{3\times 3} & tI_{3\times 3} & I_{3\times 3} \end{array}\right]$$

The adjoint operator of the state matrix X defined in (7) can be conveniently expressed as

(55) $$Ad_{X}:=\left[\begin{array}{ccc} R & 0_{3\times 3} & 0_{3\times 3}\\ v^{\wedge }R & R & 0_{3\times 3}\\ p^{\wedge }R & 0_{3\times 3} & R \end{array}\right]\in {\mathbb{R}}^{9\times 9}$$

Since (55) is a coordinate-agnostic mathematical definition, the subscripts and superscripts for **R**, ***v***, and ***p*** are omitted.

And, it exhibits a useful property [40] as follows:

(56) $$X\exp(x)X^{-1}=\exp(Ad_{X}x)$$

The right-invariant error state matrix $\eta_{k+1}^{r1}$ in (40) can be further deduced as

(57) $$\begin{array}{cc} \eta_{k+1}^{r1} & ={\hat{X}}_{k+1}X_{k+1}^{-1}\\ & =L_{\Delta t}\Phi_{\Delta t}({\hat{X}}_{k}){\hat{\Upsilon }}_{\Delta t}X_{k+1}^{-1}\\ & =L_{\Delta t}\Phi_{\Delta t}(\eta_{k}^{r1}X_{k})\Upsilon_{\Delta t}\exp{\left(-N_{\Delta k}\right)}^{-1}X_{k+1}^{-1}\\ & =L_{\Delta t}\Phi_{\Delta t}(\eta_{k}^{r1})L_{\Delta t}^{-1}\cdot L_{\Delta t}\Phi_{\Delta t}(X_{k})\Upsilon_{\Delta t}\exp{\left(-N_{\Delta k}\right)}^{-1}X_{k+1}^{-1}\\ & =\exp(Ad_{L_{\Delta t}}F_{\Delta t}x_{k}^{r1})\exp(Ad_{X_{k+1}^{r1}}N_{\Delta k}) \end{array}$$

Applying the BCH formula [44], the kinematic equation governing the prior right-invariant error in its Lie algebra form is given by

(58) xk+1r1≈−AdLΔtFΔtxkr1++AdXk+1r1NΔk

where superscript “−” denotes the prior error state, and AdLΔt=LΔtR03×303×3LΔtvLΔtR∧LΔtR03×3LΔtpLΔtR∧03×3LΔtR, $L_{\Delta t}^{R}$, $L_{\Delta t}^{v}$ and $L_{\Delta t}^{p}$ can be obtained from (29) and (40).

Obviously, another prior right-invariant error state $\eta_{k+1}^{r2}$ in (41) is the inverse of $\eta_{k+1}^{r1}$ in (40). Therefore, the error equations on the Lie group and Lie algebra can be given as

(59) $$\eta_{k+1}^{r2}=\exp(-Ad_{X_{k+1}^{r2}}N_{\Delta k})\exp(-Ad_{L_{\Delta t}}F_{\Delta t}x_{k}^{r2})$$

(60) xk+1r2≈−−AdLΔtFΔtxkr2−AdXk+1r2NΔk

Similarly, for the two left-invariant errors in (42) and (43), the kinematic equations of the prior error states and vectors in discrete time can be formulated as

(61) $$\begin{array}{cc} \eta_{k+1}^{l1} & =X_{k+1}^{-1}{\hat{X}}_{k+1}\\ & ={\left(L_{\Delta t}\Phi_{\Delta t}(X_{k})\Upsilon_{\Delta t}\right)}^{-1}L_{\Delta t}\Phi_{\Delta t}({\hat{X}}_{k}){\hat{\Upsilon }}_{\Delta t}\\ & =\Upsilon_{\Delta t}^{-1}\Phi_{\Delta t}{\left(X_{k}\right)}^{-1}L_{\Delta t}^{-1}L_{\Delta t}\Phi_{\Delta t}({\hat{X}}_{k}){\hat{\Upsilon }}_{\Delta t}\\ & =\exp{\left(-N_{\Delta k}\right)}^{-1}({\hat{\Upsilon }}_{\Delta t}^{-1})\Phi_{\Delta t}(X_{k}^{-1}{\hat{X}}_{k}){\hat{\Upsilon }}_{\Delta t}\\ & =\exp(N_{\Delta k})\exp(Ad_{{\hat{\Upsilon }}_{\Delta t}^{-1}}F_{\Delta t}x_{k}^{l1}) \end{array}$$

(62) xk+1l1≈−AdΥ^Δt−1FΔtxkl1++NΔk

(63) ηk+1l2=−exp(−AdΥ^Δt−1FΔtxkl2)exp(−NΔk)

(64) xk+1l2≈−−AdΥ^Δt−1FΔtxkl2−+NΔk

where $Ad_{{\hat{\Upsilon }}_{\Delta t}^{-1}}=\left[\begin{array}{ccc} {\left({\hat{\Upsilon }}_{\Delta t}^{R}\right)}^{-1} & 0_{3\times 3} & 0_{3\times 3}\\ -{\left({\left({\hat{\Upsilon }}_{\Delta t}^{R}\right)}^{-1}{\hat{\Upsilon }}_{\Delta t}^{v}\right)}^{\wedge }{\left({\hat{\Upsilon }}_{\Delta t}^{R}\right)}^{-1} & {\left({\hat{\Upsilon }}_{\Delta t}^{R}\right)}^{-1} & 0_{3\times 3}\\ -{\left({\left({\hat{\Upsilon }}_{\Delta t}^{R}\right)}^{-1}{\hat{\Upsilon }}_{\Delta t}^{p}\right)}^{\wedge }{\left({\hat{\Upsilon }}_{\Delta t}^{R}\right)}^{-1} & 0_{3\times 3} & {\left({\hat{\Upsilon }}_{\Delta t}^{R}\right)}^{-1} \end{array}\right]$, ${\hat{\Upsilon }}_{\Delta t}^{R}$, ${\hat{\Upsilon }}_{\Delta t}^{v}$, and ${\hat{\Upsilon }}_{\Delta t}^{p}$ can be obtained from (50).

Combined with (46), the discretization of the bias error state can be written as

(65) $$\delta b_{k+1}=\mathrm{Exp}(F_{b}\Delta t)\delta b_{k}+n_{b_{k}}$$

Let a new symbol ξ denote the error state containing ***x*** and δb, $\xi ={\left[\begin{array}{cc} x^{T} & \delta b^{T} \end{array}\right]}^{T}\in {\mathbb{R}}^{15\times 1}$. By combining the error state equation and (65), the propagation equations for the two right-invariant errors ($\xi^{r1}$ and $\xi^{r2}$) in the e-frame, which consider accelerometer and gyroscope bias errors, are as follows

(66) ξk+1r1≈−Akr1ξkr1+Bkr1nk′ξk+1r2≈−Akr2ξkr2+Bkr2nk′

where $A\in {\mathbb{R}}^{15\times 15}$ is the transition matrix for the propagation of adjacent error states, and $B\in {\mathbb{R}}^{15\times 12}$ is the transition matrix for the noise components, These matrices can be written as

$$\begin{array}{c} A_{k}^{r1}=\left[\begin{array}{cc} Ad_{L_{\Delta t}}F_{\Delta t} & Ad_{X_{k+1}^{r1}}G_{k}\\ 0_{6\times 9} & \mathrm{Exp}(F_{b}\Delta t) \end{array}\right],B_{k}^{r1}=\left[\begin{array}{cc} Ad_{X_{k+1}^{r1}}G_{k} & 0_{9\times 6}\\ 0_{6\times 6} & I_{6\times 6} \end{array}\right],\\ A_{k}^{r2}=\left[\begin{array}{cc} -Ad_{L_{\Delta t}}F_{\Delta t} & -Ad_{X_{k+1}^{r2}}G_{k}\\ 0_{6\times 9} & \mathrm{Exp}(F_{b}\Delta t) \end{array}\right],B_{k}^{r2}=\left[\begin{array}{cc} Ad_{X_{k+1}^{r2}}G_{k} & 0_{9\times 6}\\ 0_{6\times 6} & I_{6\times 6} \end{array}\right],\\ n_{k}^{\prime }={\left[\begin{array}{cccc} {n_{\omega }}^{T} & {n_{a}}^{T} & {n_{b_{\omega }}}^{T} & {n_{b_{a}}}^{T} \end{array}\right]}^{T}. \end{array}$$

It is clear that the error state transition matrix **A** in (66) is independent of the current trajectory, even when bias is taken into account. The covariances matrix of the propagation can be given as

(67) Pk+1r1=−Akr1Pkr1Akr1+TBkrQkrBkrTPk+1r2=−Akr2Pkr2Akr2+TBkrQkrBkrT

where $Q_{k}$ represents the noise covariance matrix.

Similarly, the left-invariant errors $\xi^{l}$ covariance models can be written as

(68) ξk+1l1≈−Akl1ξkl1+Bklnk′ξk+1l2≈−Akl2ξkl2+Bklnk′

(69) Pk+1l1=−Akl1Pkl1Akl1+TBklQklBklTPk+1l2=−Akl2Pkl2Akl2+TBklQklBklT

where the state transition matrices and noise transition matrix be expressed as

$$\begin{array}{c} A_{k}^{l1}=\left[\begin{array}{cc} Ad_{{\hat{\Upsilon }}_{\Delta t}^{-1}}F_{\Delta t} & G_{k}\\ 0_{6\times 9} & \mathrm{Exp}(F_{b}\Delta t) \end{array}\right],B_{k}^{l}=\left[\begin{array}{cc} G_{k} & 0_{9\times 6}\\ 0_{6\times 6} & I_{6\times 6} \end{array}\right],\\ A_{k}^{l2}=\left[\begin{array}{cc} -Ad_{{\hat{\Upsilon }}_{\Delta t}^{-1}}F_{\Delta t} & -G_{k}\\ 0_{6\times 9} & \mathrm{Exp}(F_{b}\Delta t) \end{array}\right] \end{array}$$

### 3.4. GNSS Observation Update

In the INS-based prediction process, we have defined four error states and their discrete-time error propagation equations. During the GNSS observation process, the right- and left-invariant positional error models can be written as

(70) zk+1=Hk+1r1ξk+1r1+−vobserezk+1=Hk+1r2ξk+1r2+−vobsere

(71) zk+1=Hk+1l1ξk+1l1+−vobserezk+1=Hk+1l2ξk+1l2+−vobsere

where $H^{\left(\cdot \right)}$ is the Jacobian matrix associated with the GNSS observation model.

(72) $$\begin{array}{c} H^{l1}=[\begin{array}{ccccc} R_{b}^{e}l^{\wedge } & 0_{3\times 3} & -R_{b}^{e}J^{l1} & 0_{3\times 3} & 0_{3\times 3} \end{array}]\\ H^{l2}=[-\begin{array}{ccccc} R_{b}^{e}l^{\wedge } & 0_{3\times 3} & R_{b}^{e}J^{l2} & 0_{3\times 3} & 0_{3\times 3} \end{array}] \end{array}$$

(73) $$\begin{array}{c} H^{r1}=[-\begin{array}{ccccc} {\left(p_{ib}^{e}+R_{b}^{e}l\right)}^{\wedge } & 0_{3\times 3} & J^{r1} & 0_{3\times 3} & 0_{3\times 3} \end{array}]\\ H^{r2}=[\begin{array}{ccccc} {\left(p_{ib}^{e}+R_{b}^{e}l\right)}^{\wedge } & 0_{3\times 3} & -J^{r2} & 0_{3\times 3} & 0_{3\times 3} \end{array}] \end{array}$$

The detailed derivations are presented in Appendix C. ***l*** is the lever arm vector. $v_{obser}^{e}$ denotes the observation noise, which satisfies $v_{obser}^{e}∼N(0,V)$. The calculation of the Kalman gain $K_{k+1}^{\left(\cdot \right)}$, error ${\xi_{k+1}^{\left(\cdot \right)}}^{+}$ and covariance ${P_{k+1}^{\left(\cdot \right)}}^{+}$ at time $t_{k+1}$ can be expressed as

(74) Kk+1(⋅)=Pk+1(⋅)−Hk+1(⋅)T(Hk+1(⋅)Pk+1(⋅)−Hk+1(⋅)+TVk+1)−1ξk+1(⋅)=+Kk+1(⋅)zk+1(⋅)Pk+1(⋅)=+(I−Kk+1(⋅)Hk+1(⋅))Pk+1(⋅)−

where superscript ^(⋅)^ represents the different error models from (40)–(43) and “+” denotes the posterior state. It should be noted that the error vector ${\xi_{k+1}^{\left(\cdot \right)}}^{+}$ includes error state and biases $\xi ={\left[\begin{array}{cc} x^{T} & \delta b^{T} \end{array}\right]}^{T}$. We can restore the true state in the e-frame, by the exponential mapping from the Lie group to Lie algebra, and the right-invariant errors are defined as

(75) Xk+1r1=ηk+1r1X^k+1r1=exp(xk+1r1)+−1X^k+1r1Xk+1r2=X^k+1r2ηk+1r2=−1exp(xk+1r2)+X^k+1r2

The definitions of two left-invariant errors are given by

(76) Xk+1l1=X^k+1l1ηk+1l1=X^k+1l1exp(xk+1l1)+−1Xk+1l2=ηk+1l2X^k+1l2=k+1−1X^k+1l2exp(xk+1l2)+

where

(77) $$\exp(x^{\left(\cdot \right)})=\left[\begin{array}{ccc} \exp(x_{\varphi }) & J^{\left(\cdot \right)}x_{v} & J^{\left(\cdot \right)}x_{p}\\ 0_{1\times 3} & 1 & 0\\ 0_{1\times 3} & 0 & 1 \end{array}\right]$$

where $\exp(x_{\varphi })=\Gamma_{0}(x_{\varphi })$ is the attitude error matrix in the Lie group; $Jx_{v}$ and $Jx_{p}$ represent the velocity error and position error, respectively; and $J^{\left(\cdot \right)}$ is the left Jacobian matrix, and $J^{\left(\cdot \right)}=\Gamma_{1}(x_{\varphi })$.

### 3.5. The New Errors Based on Multiple Invariant Errors

Based on the definitions of the four invariant error matrices in (40)–(43), multiplying $\eta_{t}^{r1}$ by $\eta_{t}^{r2}$ and $\eta_{t}^{l1}$ by $\eta_{t}^{l2}$ yields the following expression

(78) $$\begin{array}{c} \eta_{k}^{l1}\eta_{k}^{l2}≜I_{5\times 5}\\ \eta_{k}^{r1}\eta_{k}^{r2}≜I_{5\times 5} \end{array}$$

According the mapping relationship between Lie groups and Lie algebras given in (51), (78) can be mapped to the Lie algebra, resulting in an equivalent error vector identity as follows

(79) $$\begin{array}{c} x_{k+1}^{l1}≜-x_{k+1}^{l2}\\ x_{k+1}^{r1}≜-x_{k+1}^{r2} \end{array}$$

However, in practical operation, numerical errors, linearization and discretization approximations, and model mismatch prevent the theoretical symmetry (79) from holding exactly. To exploit this theoretical constraint and thus enhance estimation accuracy, we propose a lightweight post-processing correction strategy. Following each measurement update, we correct the error vector to mitigate random deviations induced by numerical errors while preserving the core filter structure. Since it is impractical to determine a priori which of $x_{k+1}^{1}$ and $x_{k+1}^{2}$ is superior, we adopt a symmetric fusion strategy: for the left-invariant error, the corrected vector $x_{k+1}^{l1*}$ retains the sign of $x_{k+1}^{l1}$ while its magnitude is set to the weighted average of their absolute values,

(80) xk+1l1=*sign(xk+1l1)+⊙wxk+1l1++(1−w)xk+1l2+

where sign(⋅) denotes the sign function, ⊙ represents the element-wise multiplication operator, and w is a user-defined weight coefficient (here set to w = 0.5). As the correction magnitude is typically much smaller than the uncertainty range encoded in the covariance matrix **P** (experimentally, $\left\|{x_{k+1}^{l1}}^{*}\right\|/P_{k+1}$ averages about 0.08), its impact on covariance consistency is negligible.

The core significance of this method is that it converts the theoretical symmetry of left-invariant errors (79) into a lightweight, practical correction tool. By fusing the magnitude information of both errors, we effectively curb random deviations without modifying the core filter structure, leading to improved long-term stability and accuracy. Experiments show that this lightweight post-processing step markedly reduces the root-mean-square error of state estimation, underscoring the practical benefits of exploiting geometric constraints to improve filter robustness.

The proposed correction, as a lightweight post-processing step, enhances practicality while introducing inherent trade-offs between optimality and computational efficiency. First, algorithmic simplicity and real-time constraints preclude a concurrent covariance update, causing a deliberate departure from the classical Kalman optimality framework and constituting a moderate compromise on theoretical rigor. Second, the method presupposes predominantly stochastic error deviations; performance degrades under pronounced systematic biases or severe dynamic mismatches. These limitations define the method’s applicability and motivate future work on approximate covariance updates or adaptive fusion weights.

Nevertheless, in the context of INS/GNSS integrated estimation under large misalignment angles, the corrected error vector remains subject to the inherent limitations of left-invariant errors. Specifically, the observation matrix **H***^l^* defined in (72) contains the attitude matrix $R_{b}^{e}$. This introduces attitude-induced errors into the position and velocity estimates and consequently degrades the estimation performance under large misalignment conditions. Although the multiplicative right-IEKF generally exhibits lower overall accuracy and robustness compared to the multiplicative left-IEKF [40], under large misalignment angles, it demonstrates faster convergence speed and higher estimation accuracy for position and velocity during the initial stage. As the matrix **H***^r^* (73) does not contain the attitude matrix, thereby mitigating the influence of attitude errors caused by large misalignments. Based on this analysis, this study further combines the advantages of the corrected left-invariant error ${x_{k+1}^{l1}}^{*}$ and the right-invariant error $x_{k+1}^{r1}$ to propose a multiplicative federated IEKF (MF-IEKF). The new method first employs the multiplicative right_1_-IEKF in the initial phase and then switches to the multiplicative corrected left-IEKF (MCL-IEKF) to continue the task. The federated invariant error can be written as

(81) xk+1F(i)=xk+1r1(i)tk+1<tswitchxk+1l1(*i)tk+1>tswitch

where $t_{switch}$ denotes the switching time, also called the degradation time of MR_1_-IEKF, and is set to 10 s in this study. Although MR_1_-IEKF excels at the outset, ML-IEKF achieves superior performance over the entire duration. Consequently, the optimal switching instant coincides with the crossover point where both estimators exhibit equivalent accuracy. The choice of $t_{switch}$ in our experiments resulted from a comprehensive trade-off and is not necessarily the global optimum; this choice does not undermine the demonstrated superiority of MF-IEKF.

Finally, the MF-IEKF for the INS/GNSS can be expressed as Algorithm 1.
**Algorithm 1:** MF-IEKF for the INS/GNSS**Input:** X0Rb,e,0vib,e,0pibe,0, $\left(b_{\omega ,0},b_{a,0}\right)$, $\left({\tilde{\omega }}_{ib,k}^{b},{\tilde{a}}_{ib,k}^{b}\right)$, $P_{0}$**Output:** XkRb,e,kvib,e,kpibe,k.1: **1. Initialization:**2: $X_{0}=E\left[\left(X_{0}\right)\right]$, $P_{0}=E\left[\left(X_{0}-{\hat{X}}_{0}\right){\left(X_{0}-{\hat{X}}_{0}\right)}^{T}\right]$
3: **2. State prediction at** $t_{k+1}$**:**4:   Priori invariant error and covariance propagation equation:5:   ξk+1(⋅)≈−Ak(⋅)ξk(⋅)+Bk(⋅)nk′ 6:   Pk+1(⋅)=−Ak(⋅)Pk(⋅)Ak(⋅)+TBk(⋅)Qk(⋅)Bk(⋅)T
7:   $A_{k+1}^{\left(\cdot \right)},B_{k+1}^{\left(\cdot \right)}$ matrices can be refer to Equations (66) and (68).8: **3. State update at**
$t_{k+1}$:9:    While GNSS observation arrive:10:    Kalman gain, posteriori invariant error and covariance calculation:11:   Kk+1(⋅)=Pk+1−Hk+1(⋅)T(Hk+1(⋅)Pk+1(⋅)−Hk+1(⋅)+TVk+1)−1 12:   ξk+1(⋅)=+Kk+1(⋅)zk+1(⋅) 13:   Pk+1(⋅)=+(I−Κk+1(⋅)Hk+1(⋅))Pk+1(⋅)−
14:   If $t_{k+1}<t_{switch}$, process sub-filter MR_1_-IEKF, the error calculation:15:   ξk+1=+ξk+1r1+
16:   else, process sub-filter MCL-IEKF, the error calculation:17:   ξk+1=+ξk+1l1*=+xk+1l1*δbk+1T
18:   $x_{k}^{l1*}$ can be refer to Equation (80).19: **4. Global state update:**20:   If $t_{k+1}<t_{switch}$, Xk+1=Xk+1r1=exp(xk+1r1)+−1X^k+1r1
21:   else Xk+1=Xk+1l1=X^k+1l1exp(xk+1l1*)+−1
22:   return $X_{k+1}$, ${P_{k+1}^{\left(\cdot \right)}}^{+}$
23:   End while

## 4. Experiments

This section evaluates the accuracy of the algorithms based on different error models through INS/GNSS simulation experiments and open source dataset experiments. A comparison was conducted among the following five algorithms:

The method based on the right- invariant error defined in (40), denoted as MR_1_-IEKF.

The method based on the left-invariant error defined in (58) is denoted as ML_1_-IEKF, which corresponds to the framework presented in [42].

The method based on the left-invariant error defined in (62), denoted as ML_2_-IEKF.

The method based on the corrected left-invariant error defined in (80), denoted as MCL-IEKF.

The method federated invariant error defined in (81), denoted as MF-IEKF. Note that the MCL-IEKF is a sub-filter of the MF-IEKF.

### 4.1. Numerical Simulations

**Figure 2:**
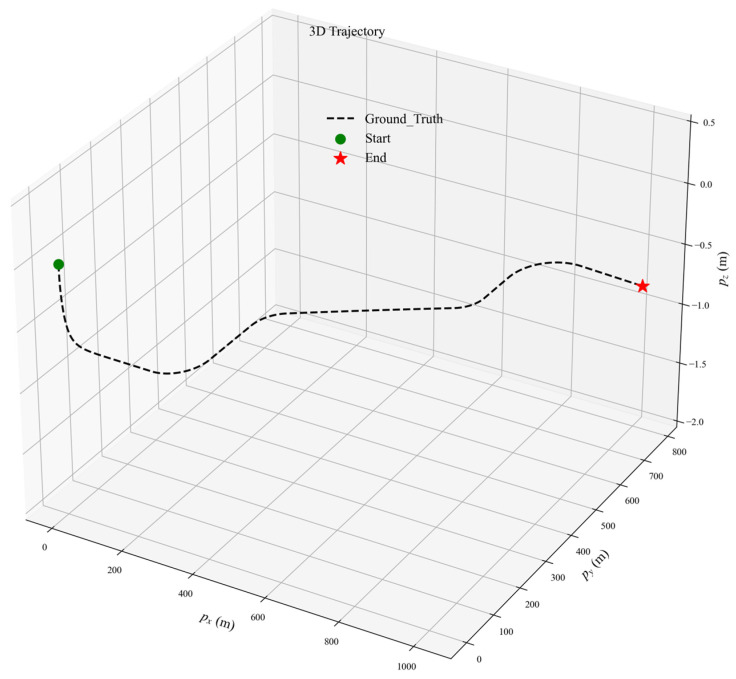
Reference trajectory for Monte Carlo experiments.

IMU and GNSS data used in the simulations were generated by the open-source platform gnss-ins-sim [45]. The toolbox supports high-, medium-, and low-grade IMU configurations, together with a low-precision GNSS receiver model. To balance the validity of algorithm performance evaluation with the reasonableness of simulation conditions, a medium-grade IMU model was selected for this study. Its parameters are specified as follows: a gyroscope bias of 3.5°/h and an angular random walk of 0.25${}^{\circ}/\sqrt{h}$; an accelerometer bias of 5 mg and a velocity random walk of 3 $\mathrm{mg}/\sqrt{\mathrm{Hz}}$. A low-precision GNSS receiver was simulated with positional noise standard deviations of σ = (5, 5, 7) m. The INS and GNSS output rates are 100 Hz and 10 Hz, respectively. The initial plat-form misalignment angles were set to [30°, 30°, 10°]. Figure 2 illustrates the reference trajectory, which includes acceleration, constant-speed cruising, and turning segments to emulate realistic vehicle dynamics.

To obtain statistically meaningful results, 500 independent Monte Carlo trials were conducted. Each trial lasted 105 s, sufficient to capture the full transition from large initial misalignment to filter convergence. All estimators shared identical initial conditions: a zero error-state vector and an identity covariance matrix.

To quantify the estimation accuracy of the algorithms in position, velocity, and attitude, the root mean square error (RMSE) was calculated, respectively, using the following formula

(82) $$\mathrm{RMSE}(k)=\sqrt{\frac{1}{n}\sum_{i=1}^{n}{\left(y_{k,i}-{\hat{y}}_{k,i}\right)}^{2}}$$

where *k* denotes the time step, *n* is the number of Monte Carlo simulations, *n* = 500, and $y_{k,i}$ ${\hat{y}}_{k,i}$ represent the reference value and estimated value in the *i*-th simulation at *k*-th time step, respectively.

**Figure 3:**
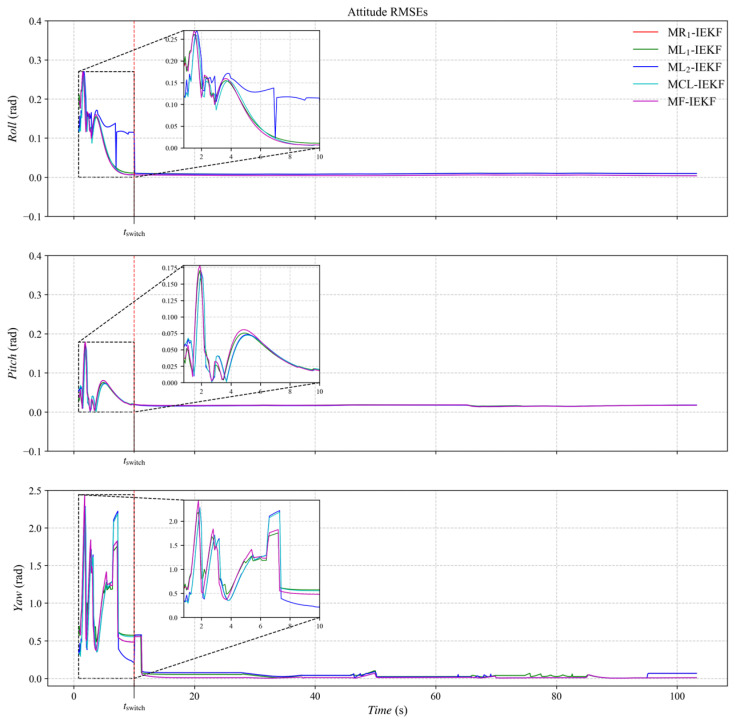
The RMSE of attitude estimation.

**Figure 4:**
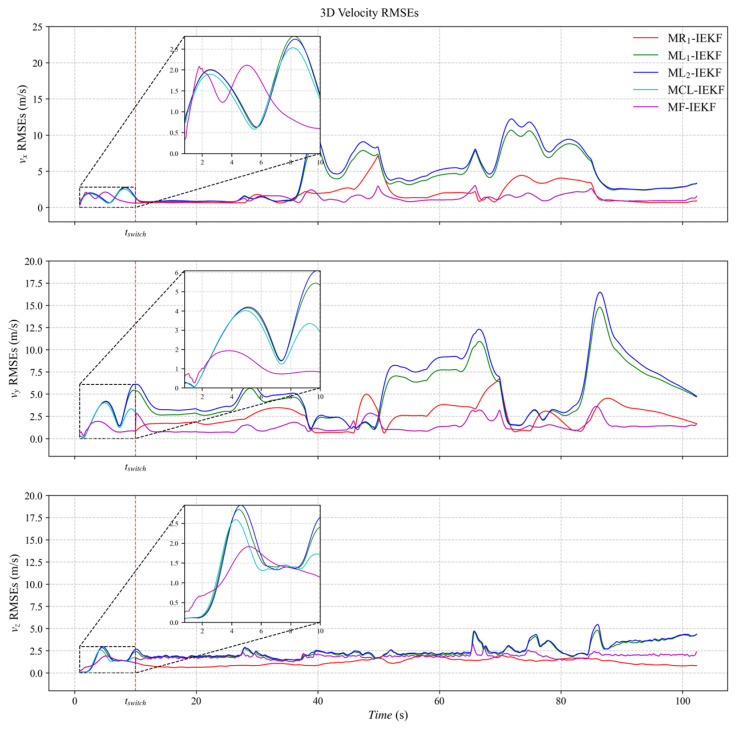
The RMSE of velocity estimation.

**Figure 5:**
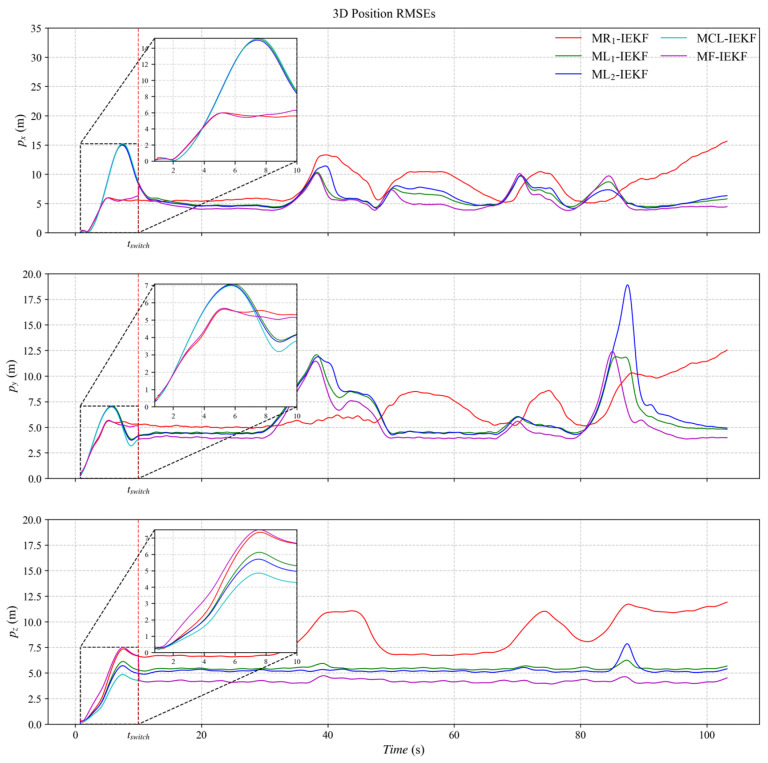
The RMSE of position estimation.

Figure 3, Figure 4 and Figure 5 present the three-axis RMSE curves for attitude, velocity, and position, respectively, obtained from the five algorithms over 500 Monte Carlo runs. As shown in Figure 3, the attitude RMSE distributions exhibit minor variations across the algorithms, requiring further quantitative analysis. Figure 4 reveals that the velocity RMSE curves of the two ML_1_- and ML_2_-IEKF are significantly higher overall than that of MCL-IEKF and MF-IEKF. Magnified views of the y- and z-axes clearly indicate that the RMSE of MCL-IEKF, which combines two ML-IEKFs, is noticeably higher than that of MF-IEKF, which incorporates MR_1_-IEKF. The position RMSE distributions in Figure 5 further support these findings. In the enlarged views of the x- and y-axes, the RMSE of MCL-IEKF remains markedly higher than that of MF-IEKF. Moreover, the magnified sections in Figure 4 and Figure 5 demonstrate that both MR_1_-IEKF and MF-IEKF achieve significantly faster convergence in velocity and position estimation compared to the ML-IEKF-based methods, which aligns with the theoretical analysis in Section 3 of the paper.

To facilitate a direct quantification and comparison of algorithm performance, the mean of RMSEs (MRMSE) is also computed as follows

(83) $$\mathrm{MRMSE}=\sqrt{\frac{1}{n}\sum_{k=1}^{n}\left(\frac{1}{3}\sum_{i=1}^{3}{\left({\mathrm{RMSE}}_{i}(k)\right)}^{2}\right)}$$

where *n* denotes the total time step, and *i* = 1, 2, 3 corresponding to the X-Y-Z axes, respectively.

The MRMSE values in Table 1 quantitatively confirm this qualitative assessment. The proposed MF-IEKF achieves superior accuracy across all metrics. As a sub-filter, the MCL-IEKF itself demonstrates significant improvements over the baseline ML_1_-IEKF, with MRMSE reductions of 13.15% in attitude, 62.01% in velocity, and 13.28% in position. Furthermore, the complete MF-IEKF framework, which incorporates MR_1_-IEKF, achieves additional performance gains, ultimately reducing attitude, velocity, and position errors by 13.86%, 63.43%, and 15.28%, respectively, compared to the ML_1_-IEKF baseline.

### 4.2. Open Source Dataset Experiments

To validate the proposed algorithm under real-world conditions, the widely used KITTI odometry dataset [46] was employed. The data were collected by a vehicle equipped with an OXTS RT3003 high-precision integrated navigation system, which provides time-synchronized IMU (100 Hz) and GNSS (10 Hz) measurements. The associated sensor noise parameters are as follows: gyroscope bias of 1.5°/h and angular random walk of 0.15${}^{\circ}/\sqrt{h}$; accelerometer bias of 0.1 mg and velocity random walk of 0.05 $\mathrm{mg}/\sqrt{\mathrm{Hz}}$; GNSS position measurement noise standard deviations of (0.02, 0.02, 0.05) m.

**Figure 6:**
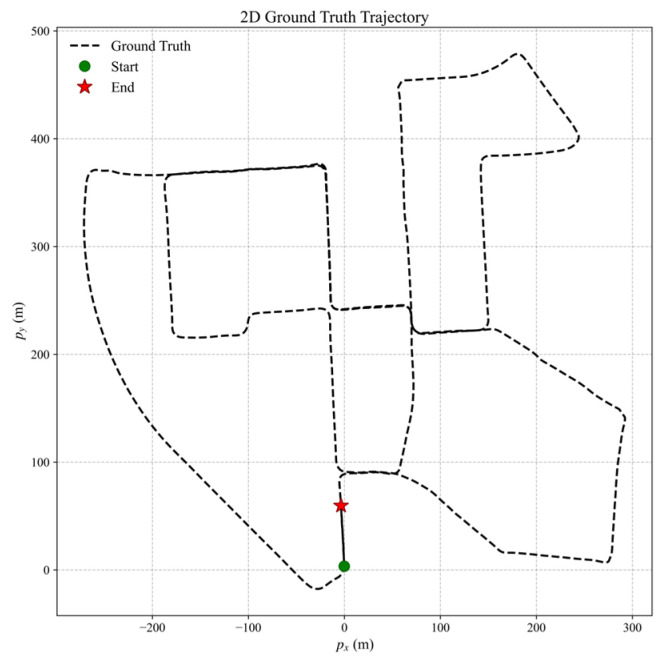
Ground truth trajectory for open source dataset experiments.

Sequence 2011_10_03_drive_0027 (duration ≈ 8 min) was selected for evaluation. This sequence covers typical urban, residential, and suburban driving environments, presenting rich motion dynamics and high representativeness; its trajectory is illustrated in Figure 6. To ensure a fair comparison, all filters were initialized under identical conditions: a zero error-state vector and an identity covariance matrix. The state update cycle was strictly synchronized with the timestamps of the dataset.

(84) $$\mathrm{AE}(k)=\left|y_{k}-{\hat{y}}_{k}\right|$$

**Figure 7:**
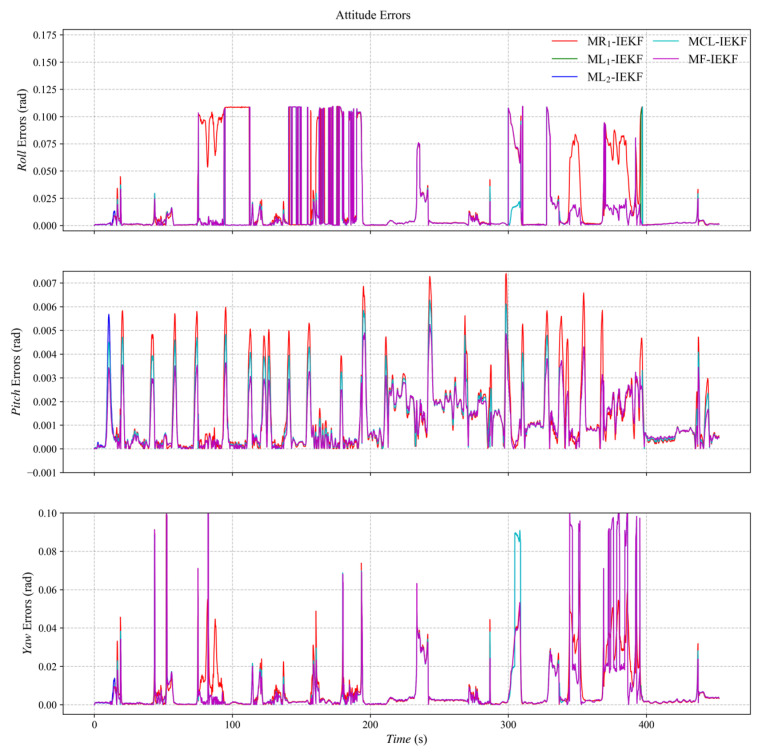
The absolute error of attitude estimation.

**Figure 8:**
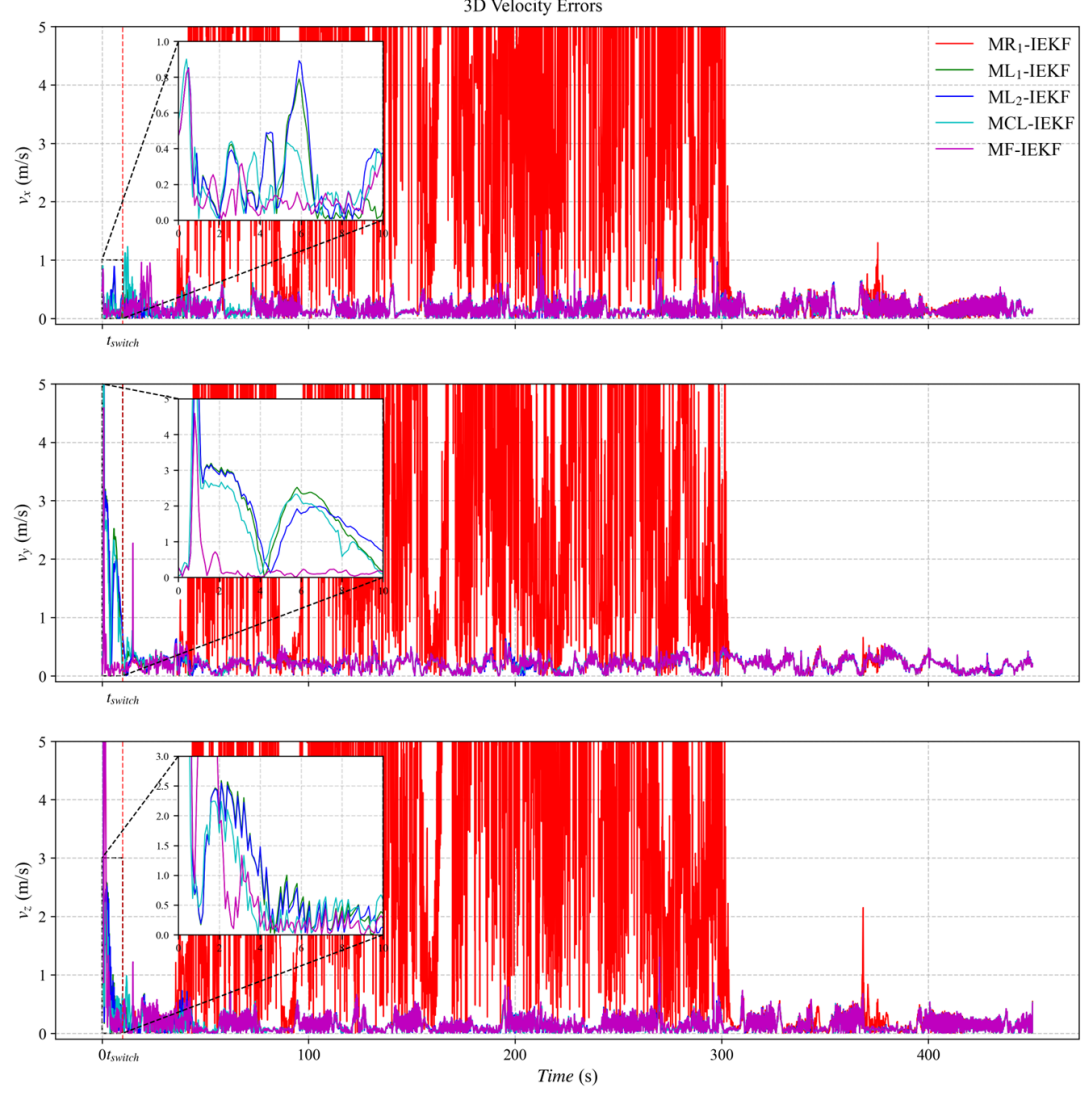
The absolute error of velocity estimation.

**Figure 9:**
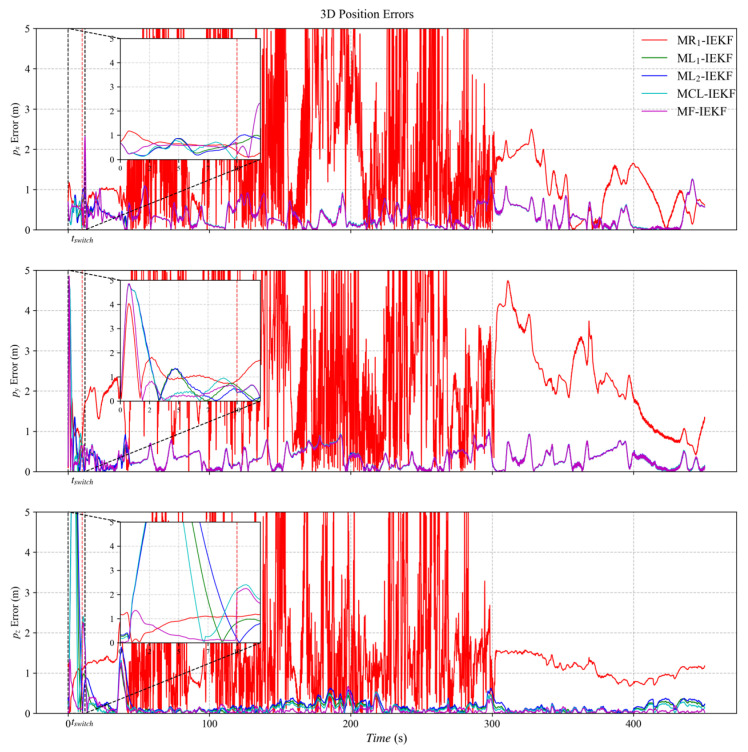
The absolute error of position estimation.

Figure 6 depicts the ground truth trajectory used in the dataset-based experiments. Figure 7, Figure 8 and Figure 9 present the absolute error (AE) distribution curves, as defined in (84), for attitude, velocity, and position estimates across the evaluated algorithms. As observed in Figure 7, the MF-IEKF achieves a notably lower AE in pitch angle compared to the other methods. The magnified view of velocity estimates in Figure 8 further demonstrates that the MF-IEKF maintains the lowest AE throughout the entire time period. Similarly, the enlarged section of position estimates in Figure 9 reveals a consistent trend, confirming that MF-IEKF has a faster convergence speed in the initial stage compared to ML-IEKF.

It should be noted that the sensors used in this dataset experiment exhibit high precision, which enables both ML_1_-IEKF and ML_2_-IEKF to also achieve reasonably accurate estimates. Under these conditions, the performance advantage of MCL-IEKF and MF-IEKF remains relatively limited outside the regions highlighted in the magnified views. Therefore, to enable an objective assessment of algorithm performance, a quantitative comparison based on the RMSE is necessary. The RMSE is calculated using the following formula

(85) $$\mathrm{RMSE}=\sqrt{\frac{1}{3}\sum_{i=1}^{3}\left(\frac{1}{n}\sum_{k=1}^{n}{\left(y_{i,k}-{\hat{y}}_{i,k}\right)}^{2}\right)}$$

where *n* denotes the total time step, $y_{k}$ and ${\hat{y}}_{k}$ represent the reference value and estimated value at *k*-th time step, and *i* = 1, 2, 3 corresponding to the X-Y-Z axes, respectively.

The RMSE results are summarized in Table 2. Compared to the baseline ML_1_-IEKF, the MCL-IEKF sub-filter achieves reductions in RMSE of 5.26% in attitude, 5.81% in velocity, and 5.98% in position. Building upon MCL-IEKF, the MF-IEKF delivers further enhancements, reducing the RMSE by an additional 3% in velocity and 31.63% in position relative to MCL-IEKF. This improvement is attributed to the incorporation of MR_1_-IEKF results during the initial phase. Notably, the MR_1_-IEKF exhibits markedly greater errors in velocity and position estimation. This stems from its inherent alignment with a local coordinate frame, in contrast to the ML-IEKF, which is naturally suited to the global frame of GNSS observations [42]. This performance disparity is further amplified under high-dynamic conditions. Overall, compared to ML_1_-IEKF, the MF-IEKF achieves total error reductions of 5.26% in attitude, 8.72% in velocity, and 35.69% in position. These results conclusively demonstrate the effectiveness of the proposed federated framework.

### 4.3. Summary and Analysis of Experiment Results

This section presents a comprehensive evaluation of five IEKF-based algorithms through rigorous numerical simulations and real-world dataset validation. The proposed MF-IEKF demonstrates superior performance in both controlled Monte Carlo simulations and KITTI dataset experiments. In simulated environments with large initial misalignments, the framework achieves statistically significant improvements in velocity and position estimation accuracy and convergence speed, attributable to its synergistic use of invariant filters. These quantitative advantages are further validated in real-world scenarios using the KITTI dataset, where MF-IEKF maintains excellent performance even under high-precision sensor conditions. This consistency verifies the algorithm’s effectiveness across different platforms and noise characteristics. The experimental results collectively confirm that the federated architecture, which strategically leverages both left- and right-invariant error models, effectively enhances the precision and reliability of INS/GNSS integrated navigation in challenging scenarios.

## 5. Conclusions

An MF-IEKF framework is proposed for INS/GNSS integration. It enhances estimation accuracy compared to baseline ML_1_-IEKF methods, demonstrating faster convergence in velocity and position estimation, particularly under large misalignment angles. The framework is built upon multiplicative pre-integration theory and introduces two core contributions: First, it operates ML_1_-IEKF and ML_2_-IEKF in parallel, leveraging the inverse relationship between their left-invariant error vectors mapped onto the Lie algebra to perform collaborative error correction. This yields the MCF-IEKF sub-filter, which improves steady-state accuracy. Second, during the initial phase, it incorporates an MR_1_-IEKF sub-filter to effectively suppress velocity and position errors induced by large misalignment angles, significantly enhancing both the convergence speed and accuracy of velocity and position estimates throughout the initial alignment process. Extensive Monte Carlo simulations and experiments on public datasets demonstrate that compared to the conventional ML_1_-IEKF, our proposed MF-IEKF framework achieves accuracy improvements of at least 5.26% in attitude, 8.72% in velocity, and 15.28% in position estimation, confirming its effectiveness and superiority in complex dynamic scenarios, particularly under large initial misalignment conditions. This research provides a viable solution for high-performance inertial-integrated navigation, and future work will focus on deeper integration of this framework with multi-source sensors (such as vision and LiDAR) and further theoretical analysis of the convergence and stability of the federated architecture.

## Figures and Tables

**Table 1:** The MRMSEs of state in the Monte Carlo experiments.

	MR_1_-IEKF	ML_1_-IEKF	ML_2_-IEKF	MCL-IEKF	MF-IEKF
Attitude (rad)	0.077	0.076	0.074	0.066	0.065
velocity (m/s)	2.012	4.186	4.660	1.590	1.530
position (m)	7.67	5.809	5.911	5.037	4.921

**Table 2:** The RMSEs of state in the open source dataset experiments.

	MR_1_-IEKF	ML_1_-IEKF	ML_2_-IEKF	MCL-IEKF	MF-IEKF
Attitude (rad)	0.026	0.019	0.019	0.018	0.018
velocity (m/s)	9.380	0.344	0.342	0.324	0.314
position (m)	4.865	1.003	1.069	0.943	0.642

## Data Availability

The raw data supporting the conclusions of this article will be made available by the authors upon request.

## Appendix A

Between the discrete time instants $t_{k}$ and $t_{k+1}$, both the Earth’s angular velocity expressed $\omega_{ie}^{e}$ in the e-frame and the IMU measurements are modeled as constant. The solution of the differential equations in (20) and (22) yields the discretized global and local increments. For the global increment, the recursive equations of global attitude, velocity and position can be obtained as follows

(A1) $$\begin{array}{l} L_{t_{k+1}}^{R}=\int_{t_{k}}^{t_{k+1}}{\left(-\omega_{ie}^{e}\right)}^{\wedge }d\tau L_{t_{k}}^{R}=\exp(-\omega_{ie}^{e}\Delta t)L_{t_{k}}^{R}\\ L_{t_{k+1}}^{v}=\exp(-\omega_{ie}^{e}\Delta t)L_{t_{k}}^{v}+\int_{0}^{\Delta t}\exp\left(-\omega_{ie}^{e}(\tau )\right)G_{ib}^{e}d\tau \\ L_{t_{k+1}}^{p}=\exp(-\omega_{ie}^{e}\Delta t)(L_{t_{k}}^{p}+\Delta tL_{t_{k}}^{v})+\int_{0}^{\Delta t}\tau \exp(-\omega_{ie}^{e}\tau )G_{ib}^{e}d\tau \end{array}$$

where the time step is $\Delta t=t_{k+1}-t_{k}$.

Over the time step Δt, the global increment can be written as

(A2) $$\begin{array}{c} L_{\Delta t}^{R}=\int_{t_{k}}^{t_{k+1}}{\left(-\omega_{ie}^{e}\right)}^{\wedge }d\tau =\exp(-\omega_{ie}^{e}\Delta t)=\Gamma_{0}\left(-\omega_{ie}^{e}\Delta t\right)\\ L_{\Delta t}^{v}=\int_{0}^{\Delta t}\exp\left(-\omega_{ie}^{e}(\tau )\right)G_{ib}^{e}d\tau =\Gamma_{1}\left(-\omega_{ie}^{e}\Delta t\right)\Delta tG_{ib}^{e}\\ L_{\Delta t}^{p}=\int_{t_{k}}^{t_{k+1}}(t_{k}-\tau )\exp(-\omega_{ie}^{e}(t_{k}-\tau ))G_{ib}^{e}d\tau =\Gamma_{2}\left(-\omega_{ie}^{e}\Delta t\right)\Delta t^{2}G_{ib}^{e} \end{array}$$

where $\Gamma_{m}(\varphi )$ was defined in (30).

For the local increment, the recursive equations of local attitude, velocity and position can be obtained as follows

(A3) $$\begin{array}{cc} \Upsilon_{t_{k+1}}^{R} & =\Upsilon_{t_{k}}^{R}\exp(\omega_{ib}^{b}\Delta t)\\ \Upsilon_{t_{k+1}}^{v} & =\Upsilon_{t_{k}}^{v}+\int_{t_{k}}^{t_{k+1}}\exp(\omega_{ib}^{b}\tau )a_{ib}^{b}d\tau \\ & =\Upsilon_{t_{k}}^{v}+\Upsilon_{t_{k}}^{R}\int_{0}^{\Delta t}\exp\left(\omega_{ib}^{b}\tau \right)d\tau a_{ib}^{b}\\ \Upsilon_{k+1}^{p} & =\Upsilon_{k}^{p}+\Delta t\Upsilon_{k}^{v}+\Upsilon_{t_{k}}^{R}\int_{0}^{\Delta t}\tau \exp(\omega_{ib}^{b}\tau )a_{ib}^{b}d\tau \end{array}$$

The local increment of discrete time can be written as

(A4) $$\begin{array}{c} \Upsilon_{\Delta t}^{R}=\exp(\omega_{ib}^{b}\Delta t)=\Gamma_{0}\left(\omega_{ib}^{b}\Delta t\right)\\ \Upsilon_{\Delta t}^{v}=\int_{0}^{\Delta t}\exp\left(\omega_{ib}^{b}\tau \right)d\tau a_{ib}^{b}=\Gamma_{1}\left(\omega_{ib}^{b}\Delta t\right)\Delta ta_{ib}^{b}\\ \Upsilon_{\Delta t}^{p}=\int_{0}^{\Delta t}\tau \exp(\omega_{ib}^{b}\tau )a_{ib}^{b}d\tau =\Gamma_{2}\left(\omega_{ib}^{b}\Delta t\right)\Delta t^{2}a_{ib}^{b} \end{array}$$

## Appendix B

Based on (47), $N_{\Delta t}$ can be further written as

(A5) $$-N_{\Delta t}=-G_{k}n_{k}={\left[\begin{array}{ccc} {\hat{\varphi }}^{\left(n\right)} & {\hat{v}}^{\left(n\right)} & {\hat{p}}^{\left(n\right)} \end{array}\right]}^{T}\in {\mathbb{R}}^{9\times 1}$$

where the Jacobian matrix $G_{k}\in {\mathbb{R}}^{9\times 6}$ can be written as

(A6) $$G_{k}=\left[\begin{array}{cc} G_{11} & G_{12}\\ G_{21} & G_{22}\\ G_{31} & G_{32} \end{array}\right]$$

The exponential form of $N_{\Delta t}$ is

(A7) $$\exp(-N_{\Delta t})=\left[\begin{array}{ccc} \exp({\hat{\varphi }}^{\left(n\right)}) & \hat{J}{\hat{v}}^{\left(n\right)} & \hat{J}{\hat{p}}^{\left(n\right)}\\ 0_{1\times 3} & 1 & 0\\ 0_{1\times 3} & 0 & 1 \end{array}\right]$$

where $\exp({\hat{\varphi }}^{\left(n\right)})=\Gamma_{0}({\hat{\omega }}_{ib}^{b}\Delta t)$, $\Gamma_{m}(\varphi )$ was defined in (30), and $\hat{J}=\Gamma_{1}({\hat{\omega }}_{ib}^{b}\Delta t)$, ${\hat{\omega }}_{ib}^{b}={\tilde{\omega }}_{ib}^{b}-{\hat{b}}_{\omega }$.

Based on (44), the equation can be obtained as

(A8) $${\hat{\Upsilon }}_{\Delta t}^{-1}\Upsilon_{\Delta t}=\left[\begin{array}{ccc} \exp({\hat{\varphi }}^{\left(n\right)}) & \hat{J}{\hat{v}}^{\left(n\right)} & \hat{J}{\hat{p}}^{\left(n\right)}\\ 0_{1\times 3} & 1 & 0\\ 0_{1\times 3} & 0 & 1 \end{array}\right]$$

where $\Upsilon_{\Delta t}$ and ${\hat{\Upsilon }}_{\Delta t}^{-1}$ can be found in (34) and (50). $-N_{\Delta t}$ can be computed as follows

(A9) $$\begin{array}{cc} {\hat{\varphi }}^{\left(n\right)} & =\log\left(\Gamma_{0}{\left({\hat{\omega }}_{ib}^{b}\Delta t\right)}^{-1}\Gamma_{0}\left(\omega_{ib}^{b}\Delta t\right)\right)\\ & \approx \log\left(\Gamma_{0}{\left({\hat{\omega }}_{ib}^{b}\Delta t\right)}^{-1}\Gamma_{0}\left({\hat{\omega }}_{ib}^{b}\Delta t\right)\Gamma_{0}\left(\Gamma_{1}^{T}\left(\omega_{ib}^{b}\Delta t\right)\left(-n_{\omega }\Delta t\right)\right)\right)\\ & =\log\left(\Gamma_{0}\left(\Gamma_{1}^{T}\left({\hat{\omega }}_{ib}^{b}\Delta t\right)\left(-n_{\omega }\Delta t\right)\right)\right)\\ & =-\Gamma_{1}^{T}\left({\hat{\omega }}_{ib}^{b}\Delta t\right)\Delta tn_{\omega }\\ & =\Gamma_{0}^{-1}\left({\hat{\omega }}_{ib}^{b}\Delta t\right)\Gamma_{1}\left({\hat{\omega }}_{ib}^{b}\Delta t\right)\Delta tn_{\omega } \end{array}$$

where log(⋅) is the inverse of the exponential operation; the real value of angular velocity is $\omega_{ib}^{b}={\hat{\omega }}_{ib}^{b}-n_{\omega }$, and due to the noise $n_{\omega }$ is small, based on the property of $\Gamma_{m}\left(\varphi \right)$, $\Gamma_{m}\left(\varphi +\varphi^{\prime }\right)\approx \Gamma_{m}\left(\varphi \right)\Gamma_{m}\left(\Gamma_{m+1}\left(-\varphi \right)\varphi^{\prime }\right)$ [29], $\Gamma_{0}\left(\omega_{ib}^{b}\Delta t\right)$ can be written as

(A10) $$\begin{array}{c} \begin{array}{cc} {\hat{v}}^{\left(n\right)} & =\Gamma_{0}{\left({\hat{\omega }}_{ib}^{b}\Delta t\right)}^{-1}\left(\Gamma_{1}\left(\omega_{ib}^{b}\Delta t\right)a_{ib}^{b}\Delta t-\Gamma_{1}\left({\hat{\omega }}_{ib}^{b}\Delta t\right){\hat{a}}_{ib}^{b}\Delta t\right)\\ & \approx \Gamma_{0}{\left({\hat{\omega }}_{ib}^{b}\Delta t\right)}^{-1}\left(\Gamma_{1}\left({\hat{\omega }}_{ib}^{b}\Delta t\right)\Gamma_{0}\left(\Gamma_{2}^{T}\left({\hat{\omega }}_{ib}^{b}\Delta t\right)\left(-n_{\omega }\Delta t\right)\right)a_{ib}^{b}\Delta t\right.\\ & \left.-\Gamma_{1}\left({\hat{\omega }}_{ib}^{b}\Delta t\right){\hat{a}}_{ib}^{b}\Delta t\right)\\ & \approx \Gamma_{0}{\left({\hat{\omega }}_{ib}^{b}\Delta t\right)}^{-1}\left(\Gamma_{1}\left({\hat{\omega }}_{ib}^{b}\Delta t\right)a_{ib}^{b}\Delta t-\Gamma_{1}\left({\hat{\omega }}_{ib}^{b}\Delta t\right){\hat{a}}_{ib}^{b}\Delta t\right.\\ & \left.+\Gamma_{1}\left({\hat{\omega }}_{ib}^{b}\Delta t\right){\left(\Gamma_{2}^{T}\left({\hat{\omega }}_{ib}^{b}\Delta t\right)\left(-n_{\omega }\Delta t\right)\right)}^{\wedge }a_{ib}^{b}\Delta t\right)\\ & =\Gamma_{0}{\left({\hat{\omega }}_{ib}^{b}\Delta t\right)}^{-1}\left(\Gamma_{1}\left({\hat{\omega }}_{ib}^{b}\Delta t\right)\left(-n_{a}\Delta t\right)\right.\\ & \left.+\Gamma_{1}\left({\hat{\omega }}_{ib}^{b}\Delta t\right){\left(a_{ib}^{b}\right)}^{\wedge }\Gamma_{2}^{T}\left({\hat{\omega }}_{ib}^{b}\Delta t\right)\left(n_{\omega }\Delta t\right)\Delta t\right)\\ & =-\left(\left(-\Gamma_{0}{\left({\hat{\omega }}_{ib}^{b}\Delta t\right)}^{-1}\Gamma_{1}\left({\hat{\omega }}_{ib}^{b}\Delta t\right)\cdot \left({a_{ib}^{b}}^{\wedge }\right)\Gamma_{2}^{T}\left({\hat{\omega }}_{ib}^{b}\Delta t\right)\Delta t^{2}\right)n_{\omega }\right.\\ & \left.+\Gamma_{0}{\left({\hat{\omega }}_{ib}^{b}\Delta t\right)}^{-1}\Gamma_{1}\left({\hat{\omega }}_{ib}^{b}\Delta t\right)\Delta tn_{a}\right) \end{array} \end{array}$$

(A11) $$\begin{array}{cc} {\hat{v}}^{\left(n\right)} & =\Gamma_{0}{\left({\hat{\omega }}_{ib}^{b}\Delta t\right)}^{-1}\left(\Gamma_{1}\left(\omega_{ib}^{b}\Delta t\right)a_{ib}^{b}\Delta t-\Gamma_{1}\left({\hat{\omega }}_{ib}^{b}\Delta t\right){\hat{a}}_{ib}^{b}\Delta t\right)\\ & \approx \Gamma_{0}{\left({\hat{\omega }}_{ib}^{b}\Delta t\right)}^{-1}\left(\Gamma_{1}\left({\hat{\omega }}_{ib}^{b}\Delta t\right)\Gamma_{0}\left(\Gamma_{2}^{T}\left({\hat{\omega }}_{ib}^{b}\Delta t\right)\left(-n_{\omega }\Delta t\right)\right)a_{ib}^{b}\Delta t\right.\\ & \left.-\Gamma_{1}\left({\hat{\omega }}_{ib}^{b}\Delta t\right){\hat{a}}_{ib}^{b}\Delta t\right)\\ & \approx \Gamma_{0}{\left({\hat{\omega }}_{ib}^{b}\Delta t\right)}^{-1}\left(\Gamma_{1}\left({\hat{\omega }}_{ib}^{b}\Delta t\right)a_{ib}^{b}\Delta t-\Gamma_{1}\left({\hat{\omega }}_{ib}^{b}\Delta t\right){\hat{a}}_{ib}^{b}\Delta t\right.\\ & \left.+\Gamma_{1}\left({\hat{\omega }}_{ib}^{b}\Delta t\right){\left(\Gamma_{2}^{T}\left({\hat{\omega }}_{ib}^{b}\Delta t\right)\left(-n_{\omega }\Delta t\right)\right)}^{\wedge }a_{ib}^{b}\Delta t\right)\\ & =\Gamma_{0}{\left({\hat{\omega }}_{ib}^{b}\Delta t\right)}^{-1}\left(\Gamma_{1}\left({\hat{\omega }}_{ib}^{b}\Delta t\right)\left(-n_{a}\Delta t\right)\right.\\ & \left.+\Gamma_{1}\left({\hat{\omega }}_{ib}^{b}\Delta t\right){\left(a_{ib}^{b}\right)}^{\wedge }\Gamma_{2}^{T}\left({\hat{\omega }}_{ib}^{b}\Delta t\right)\left(n_{\omega }\Delta t\right)\Delta t\right)\\ & =-\left(\left(-\Gamma_{0}{\left({\hat{\omega }}_{ib}^{b}\Delta t\right)}^{-1}\Gamma_{1}\left({\hat{\omega }}_{ib}^{b}\Delta t\right)\cdot \left({a_{ib}^{b}}^{\wedge }\right)\Gamma_{2}^{T}\left({\hat{\omega }}_{ib}^{b}\Delta t\right)\Delta t^{2}\right)n_{\omega }\right.\\ & \left.+\Gamma_{0}{\left({\hat{\omega }}_{ib}^{b}\Delta t\right)}^{-1}\Gamma_{1}\left({\hat{\omega }}_{ib}^{b}\Delta t\right)\Delta tn_{a}\right) \end{array}$$

where $a_{ib}^{b}={\hat{a}}_{ib}^{b}-n_{a}$.

(A12) $$\begin{array}{cc} {\hat{p}}^{\left(n\right)} & =\Gamma_{0}{\left({\hat{\omega }}_{ib}^{b}\Delta t\right)}^{-1}\Gamma_{2}\left(\omega_{ib}^{b}\Delta t\right)a_{ib}^{b}\Delta t^{2}-\Gamma_{2}\left(\omega_{ib}^{b}\Delta t\right){\hat{a}}_{ib}^{b}\Delta t^{2}\\ & =\Gamma_{0}{\left({\hat{\omega }}_{ib}^{b}\Delta t\right)}^{-1}\left(\Gamma_{2}\left(\omega_{ib}^{b}\Delta t\right)\Gamma_{0}\left(\Gamma_{3}^{T}\left({\hat{\omega }}_{ib}^{b}\Delta t\right)\left(-n_{\omega }\Delta t\right)\right)a_{ib}^{b}\Delta t^{2}\right.\\ & \left.-\Gamma_{2}\left(-{\hat{\omega }}_{ib}^{b}\Delta t\right){\hat{a}}_{ib}^{b}\Delta t^{2}\right)\\ & \approx \Gamma_{0}{\left({\hat{\omega }}_{ib}^{b}\Delta t\right)}^{-1}\left(\Gamma_{2}\left(\omega_{ib}^{b}\Delta t\right)a_{ib}^{b}\Delta t^{2}-\Gamma_{2}\left(\omega_{ib}^{b}\Delta t\right){\hat{a}}_{ib}^{b}\Delta t^{2}\right.\\ & \left.+\Gamma_{2}\left(-{\hat{\omega }}_{ib}^{b}\Delta t\right){\left(\Gamma_{3}^{T}\left({\hat{\omega }}_{ib}^{b}\Delta t\right)\left(-n_{\omega }\Delta t\right)\right)}^{\wedge }a_{ib}^{b}\Delta t^{2}\right)\\ & =-\left(-\Gamma_{0}{\left({\hat{\omega }}_{ib}^{b}\Delta t\right)}^{-1}\Gamma_{2}\left({\hat{\omega }}_{ib}^{b}\Delta t\right){\left(a_{ib}^{b}\right)}^{\wedge }\Gamma_{3}^{T}\left({\hat{\omega }}_{ib}^{b}\Delta t\right)\Delta t^{3}n_{\omega }\right.\\ & \left.+\Gamma_{0}{\left({\hat{\omega }}_{ib}^{b}\Delta t\right)}^{-1}\Gamma_{2}\left({\hat{\omega }}_{ib}^{b}\Delta t\right)\Delta t^{2}n_{a}\right) \end{array}$$

In (A5), (A6) and (A8), since the dependent variable in function $\Gamma_{m}\left(\varphi \right)$ is ${\hat{\omega }}_{ib}^{b}\Delta t$, we omit it for brevity. The noise driven matrix $G_{k}$ is shown below as

(A13) Gk=Γ0−1Γ1Δt03×3−Γ0−1Γ1aibbΓ2T∧Δt2Γ0−1Γ1Δt−Γ0−1Γ2aibbΓ3T∧Δt3Γ0−1Γ2Δt2

## Appendix C

Based on the definition of the left-invariant error matrix given in (42) and the mapping relationship between Lie groups and Lie algebras provided in (51), the left-invariant error matrix $\eta^{l1}$ can be further expressed as

(A14) $$X^{-1}\hat{X}=\exp(x^{l1})$$

Expansion of both sides of (A14) gives

(A15) R^be=Rbeexp(xφl1)∧≈Rbe(I3×3+xφl1)∧δpibe=p^ibe−pibe=R^beJl1xpl1

The derivation of the observation error proceeds from (71) as follows

(A16) $$\begin{array}{cc} z_{k+1}^{l1} & ={\hat{p}}_{obser}^{e}-{\hat{p}}_{pred}^{e}\\ & =p_{obser}^{e}+v_{obser}^{e}-(p_{pred}^{e}+\delta p_{pred}^{e}+{\hat{R}}_{b}^{e}l)\\ & \approx (p_{obser}^{e}-p_{pred}^{e}-R_{b}^{e}l-\delta p_{pred}^{e})+R_{b}^{e}l^{\wedge }x_{\varphi }^{l1}+v_{obser}^{e}\\ & =-\delta p_{ib}^{e}+R_{b}^{e}l^{\wedge }x_{\varphi }^{l1}+v_{obser}^{e}\\ & =R_{b}^{e}l^{\wedge }x_{\varphi }^{l1}-R_{b}^{e}J^{l1}x_{p}^{l1}+v_{obser}^{e}\\ & =H^{l1}\xi^{l1}+v_{obser}^{e} \end{array}$$

where $H^{l1}=R_{b}^{e}[\begin{array}{ccccc} l^{\wedge } & 0_{3\times 3} & -J^{l1} & 0_{3\times 3} & 0_{3\times 3} \end{array}]$. According to (79), it can be readily verified that $H^{l2}=-H^{l1}$. Similarly, the Jacobian matrices **H***^r^*^1^ and **H***^r^*^2^ can also be derived, and their derivations are omitted here.
